# Supplementary material for: Human encroachment, climate change and the loss of our archaeological organic cultural heritage: Accelerated bone deterioration at Ageröd, a revisited Scandinavian Mesolithic key-site in despair
Source: PLoS One. 2020 Jul 29;15(7):e0236105. doi: 10.1371/journal.pone.0236105 (PMC7390309; doi:10.1371/journal.pone.0236105)
Supplement: S1 File — (PDF) [file pone.0236105.s001.pdf]

Supporting information for: Human encroachment, climate change and the loss of our archaeological organic cultural heritage: Accelerated bone deterioration at Ageröd, a revisited Scandinavian Mesolithic key-site in despair

Tab. S1. General information from the five 2019 trenches

**Trench**	**201**	**205**	**209**	**217**	**259**
Zone	2	3	3	2	1
Size, cm	100 x 100	100 x 100	100 x 100	100 x 100	100 x 100
Depth, cm	85	60	55	90	60
Total water sieved, ca	35%	25%	25%	35%	15%
Soil bank water sieved, ca	25%	NA	NA	25	NA
Upper peat water sieved, ca	25%	25%	25%	25%	15%
Cultural layer water sieved, ca	100%	25%	25%	100%	15%
Lower peat water sieved, ca	35%	25%	25%	35%	15%
Bone, number of specimens (NSP)	172	11	2	167	0
Total bone weight (g)	397	148	1	507	0
Burnt bones frequency	7,5%	0%	100%	3%	NA
Bone, relative quantity (NSP/m^2)	202,4	18,3	3,6	185,6	0
Bone, relative mass (g/m^2)	467,1	246,7	1,8	563,3	0
Lithic material, number of	793	172	133	499	315
Lithic material, weight (g)	1835	1603	234	1658	415
Lithic, relative quantity (NSP/m^2)	932,9	286,7	241,8	554,4	525
Lithic, relative mass (g/m^2)	2158,8	2671,7	425,5	1842,2	691,7
Comments	Drier area	Wetter area	Wetter area	Drier area	Partly dug through undocumented trench, drier area

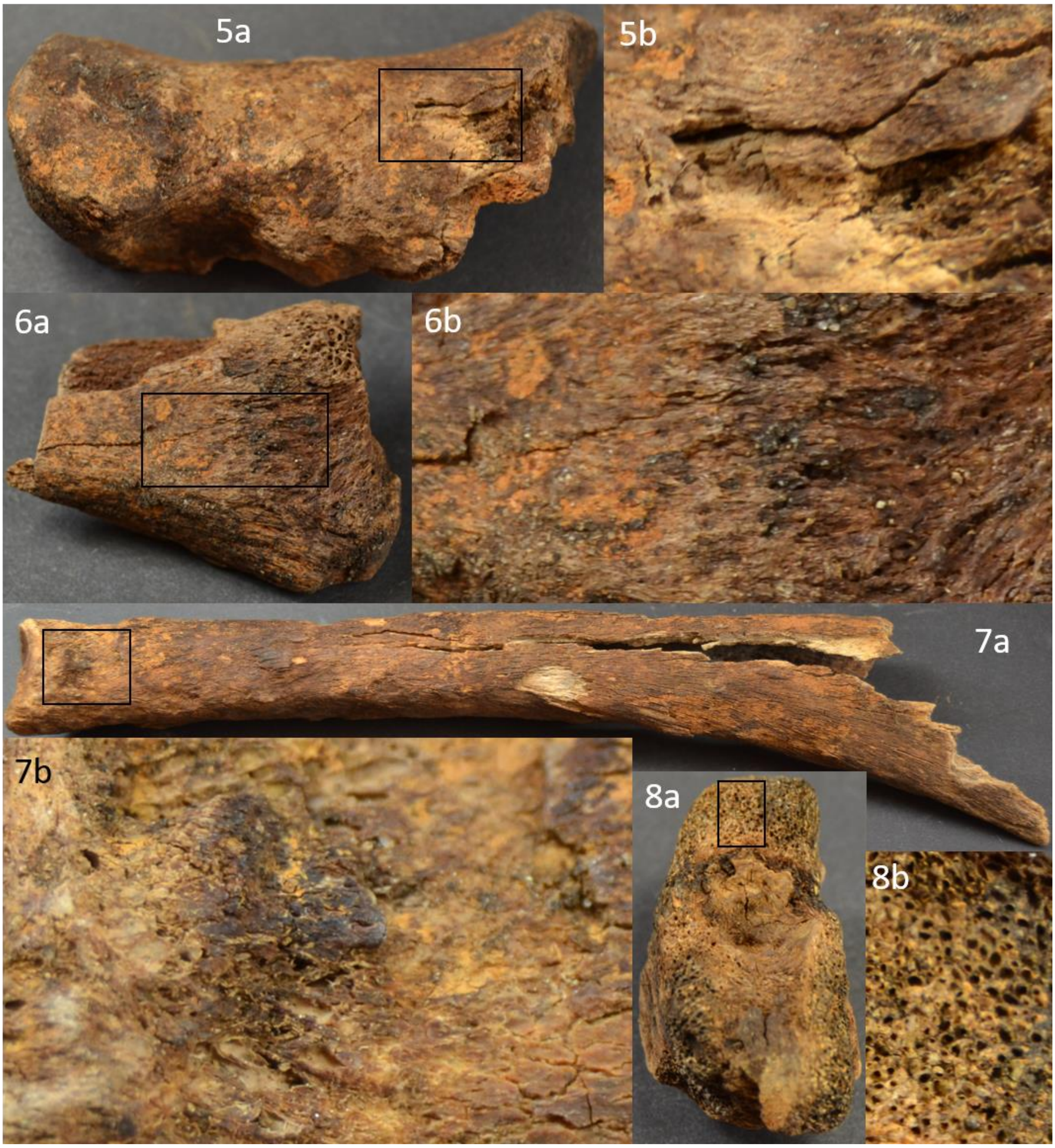

Fig. S1. Weathering II categories 5-8, a = entire bone b = zoom of boxed area: Category 5: Aurochs phalanx 1, all sides outer surface erosion less than 50%. Category 6: Elk phalanx 2, all sides outer surface erosion more than 50%. Category 7: Red deer tibia, no remaining surface, all sides average bone loss less than 5 mm. Category 8: Wild boar astragalus, no remaining surface, all sides average bone loss more than 5 mm. Image created for this publication by the authors (AB).

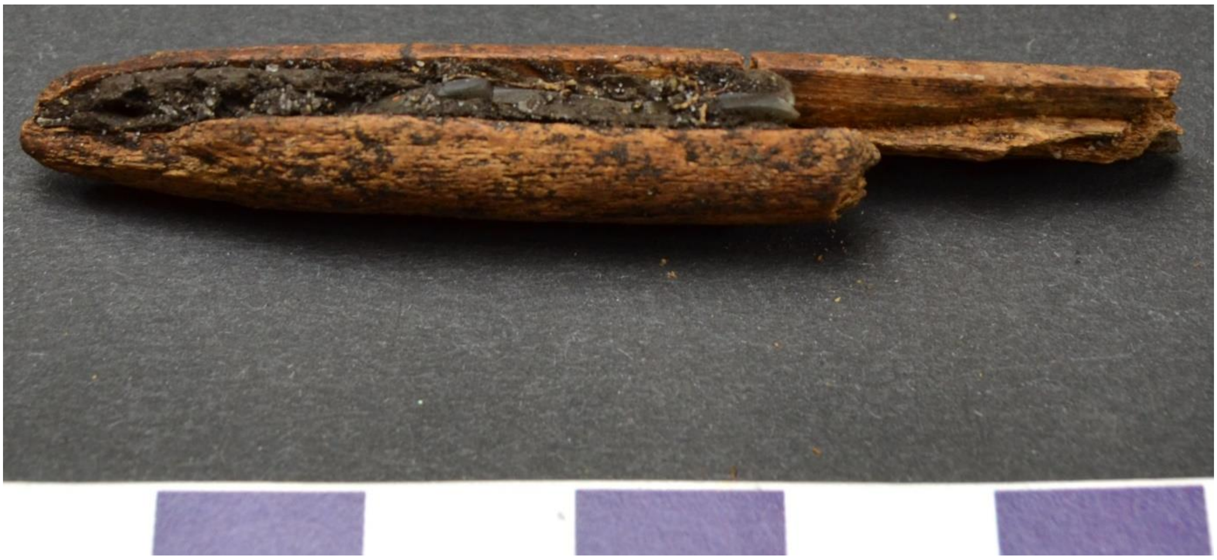

Fig. S2. Slotted bone point with resin and inserted microliths recovered in the upper part of the soil bank in trench 201. Photography created for this publication by the authors (AB).

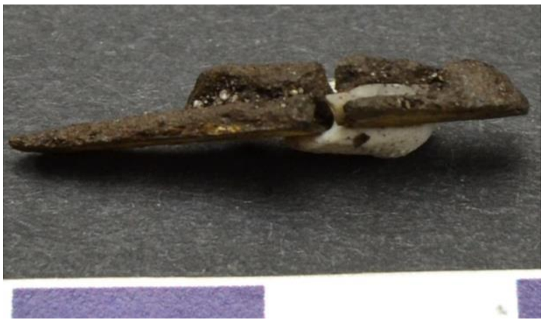

Fig. S3. Piece of resin with a microlith in the middle and bone dust on the outside, suggesting that the resin and flint piece had previously been inserted in a slotted bone point. Photography created for this publication by the authors (AB).

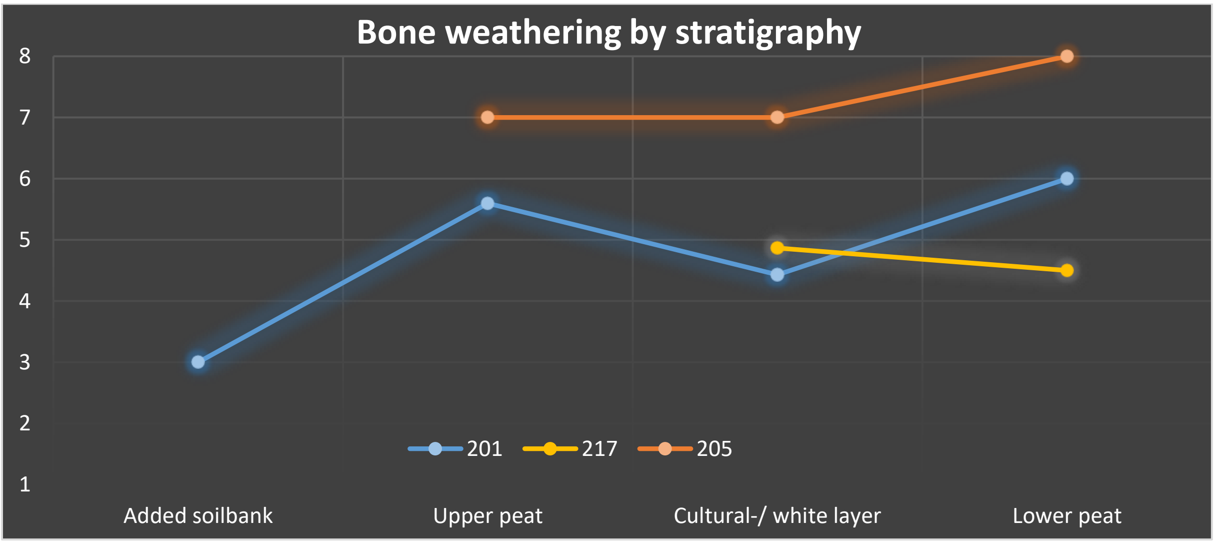

Fig. S4. Average bone weathering by stratigraphy in the three bone yielding trenches from 2019. Severity of degradation increase with higher weathering degree numbers. Weathering degrees <3 are considered low, 3 medium and 4-8 high. N: Trench 201=26; Trench 217=17; Trench 205=9.

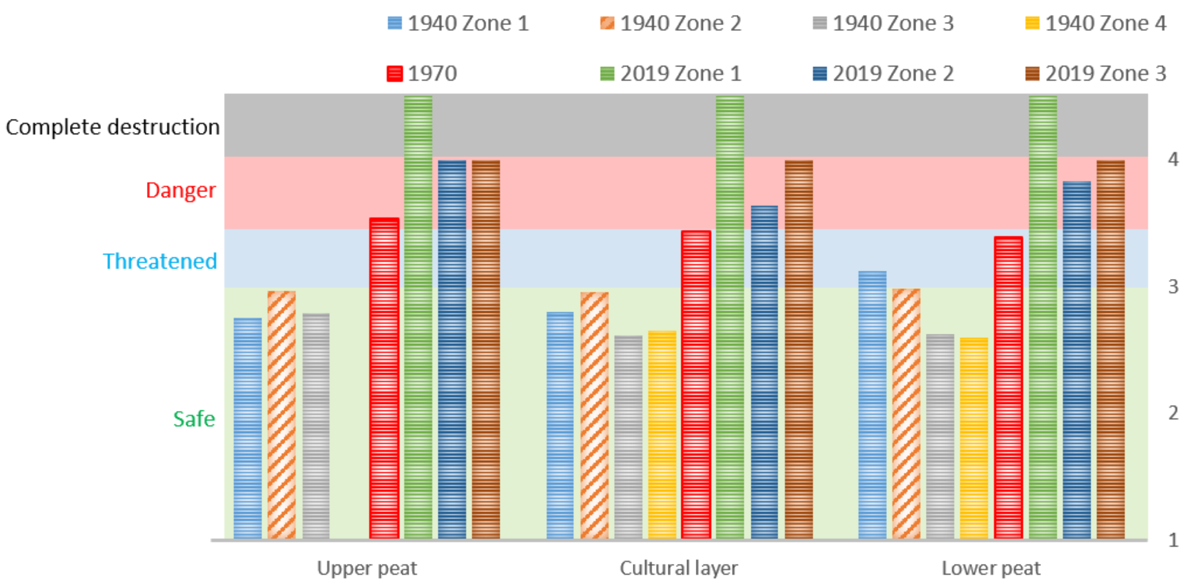

Fig. S5. Mean weathering degree on all bones that could be connected to a specific layer and a specific zone. Severity of degradation increase with higher weathering degree numbers. Weathering degrees <3 are considered low, 3 medium and 4 high. N: 1940s (zone 1=541; zone 2=590; zone 3=964; zone 4= 358); 1970s=181; 2019 (zone 2=43; zone 3=9).

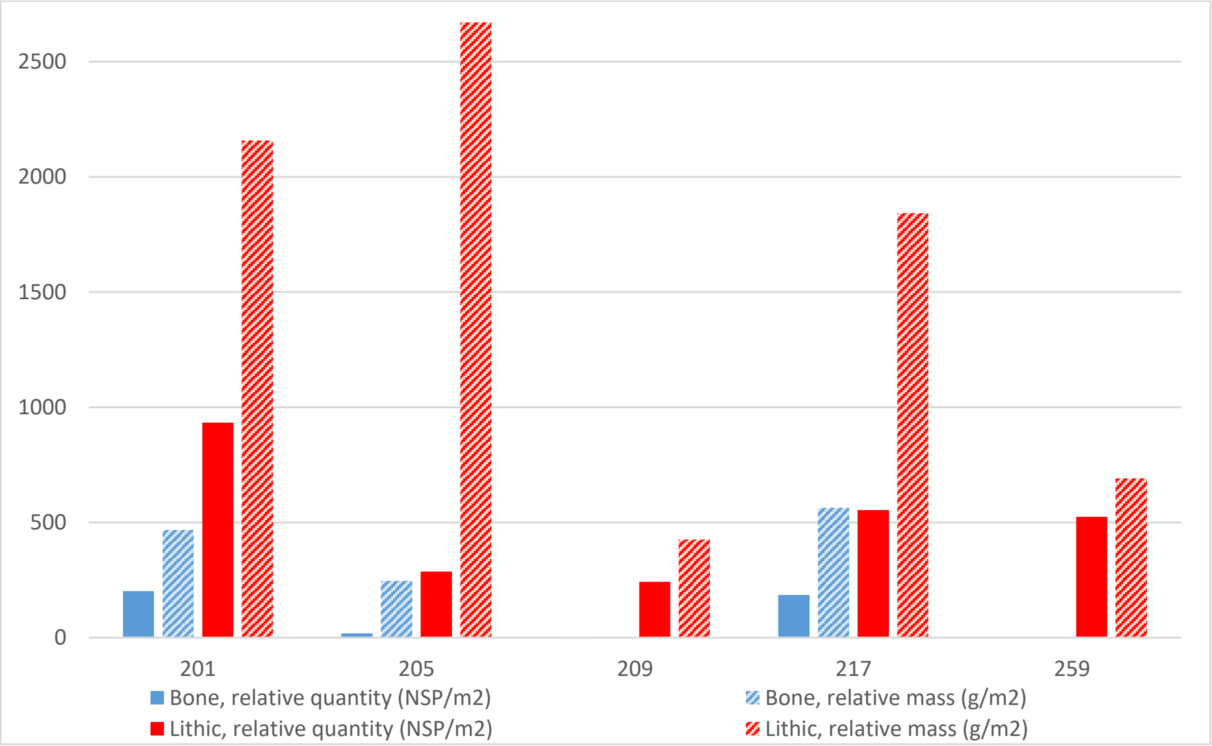

Fig. S6. The relative amount of worked flint and bone remains per m^2 in each of the 2019 trenches. Lithic remains are frequently occurring in the trenches devoid of bone material, which indicate the there are no major post-deposition disturbances or irregularities in deposition patterns which could explain why the organic material is lacking in trench 209 and 259.

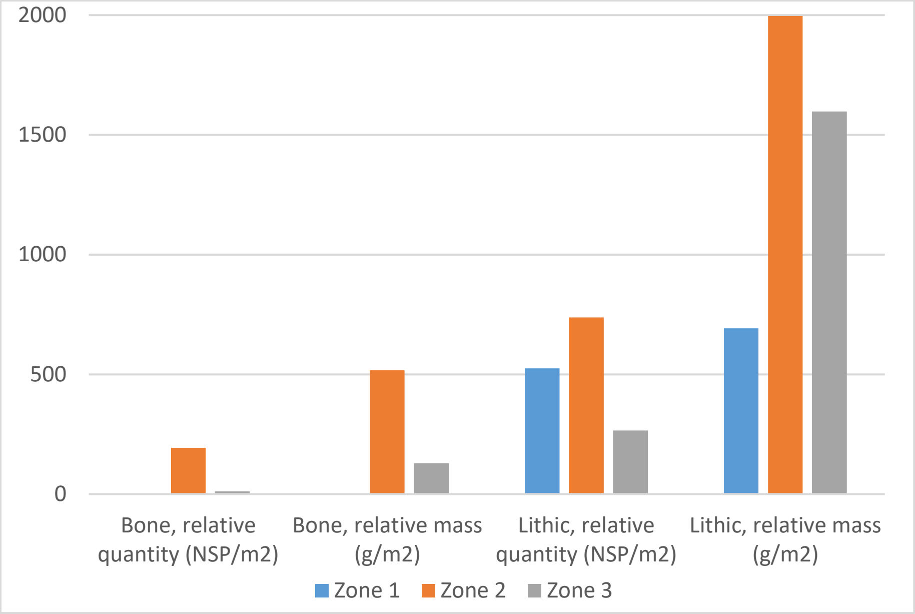

Fig. S7. The relative amount of worked flint and bone remains per m^2 *in the three zones excavated in 2019*
