# Supplementary material for: Human encroachment, climate change and the loss of our archaeological organic cultural heritage: Accelerated bone deterioration at Ageröd, a revisited Scandinavian Mesolithic key-site in despair
Source: PLoS One. 2020 Jul 29;15(7):e0236105. doi: 10.1371/journal.pone.0236105 (PMC7390309; doi:10.1371/journal.pone.0236105)
Supplement: Attachment — Submitted filename: PONE-D-20-10718_reviewer.pdf [file pone.0236105.s002.pdf]

PLOS ONE

Human encroachment, climate change and the loss of our archaeological organic cultural heritage: Accelerated bone deterioration on Ageröd, a revisited Scandinavian Mesolithic key-site in despair

--Manuscript Draft--

**Manuscript Number:**	PONE-D-20-10718
**Article Type:**	Research Article
**Full Title:**	Human encroachment, climate change and the loss of our archaeological organic cultural heritage: Accelerated bone deterioration on Ageröd, a revisited Scandinavian Mesolithic key-site in despair
**Short Title:**	Human encroachment, climate change and the loss of our archaeological organic cultural heritage
**Corresponding Author:**	Adam Boethius<br>Lunds Universitet<br>Lund, SWEDEN
**Keywords:**	Holocene; Europe; Bone deterioration; zooarchaeology; taphonomy; Mesolithic; Scandinavia; Heritage management; climate change
**Abstract:**	Ancient organic remains are essential for the reconstruction of past human lifeways and environments but are only preserved under particular conditions. Recent findings indicate that such conditions are becoming rarer and that old archaeological sites, with previous good preservation, are losing their preservative qualities. To investigate this, we returned to the well-known Swedish Mesolithic site Ageröd I. Here we present the result of the re-excavation and the osteological analyses of the bone remains from the 1940s, 1970s and 2019 excavation campaigns of the site, to document and quantify changes in bone preservation and relate them to variations in soil conditions and on-site topography. The results indicate that the bone material has suffered from accelerated deterioration during the last 75 years. In some areas, this has led to a complete destruction of the remains while other areas, while suffering badly, are still able to preserve archaeological bones. We conclude that while Ageröd can still be considered an important site, it has lost much of the properties that made it unique and if no actions are taken to secure its well-being it will soon lose the organic remains that before modern encroachment and climate change had been pristinely preserved at the site for 9000 years. Finally, because nothing special, compared to most other archaeological sites, has happened to Ageröd during these last 75 years, our results also raise questions of the state of organic preservation in other areas and call for a broad examination of our most vulnerable hidden archaeological remains.
**Order of Authors:**	Adam Boethius<br>Mathilda Kjällquist<br>Ola Magnell<br>Jan Apel
**Additional Information:**	
**Question**	**Response**
**Financial Disclosure**<br><br>Enter a financial disclosure statement that describes the sources of funding for the work included in this submission. Review the submission guidelines for detailed requirements. View published research articles from *PLOS ONE* for specific examples.	We are grateful for the financial support from:<br>JA, Crafoord foundation, nr. 20180631 https://www.crafoord.se/<br>AB, Lennart J. Hägglunds foundation for archaeological research and education http://hagglundsstiftelse.se/<br>AB, the Swedish National Heritage Board, RAÄ-2018-3237, https://www.raa.se/<br>The funders had no role in study design, data collection and analysis, decision to publish, or preparation of the manuscript.

Powered by Editorial Manager® and ProduXion Manager® from Aries Systems Corporation

This statement is required for submission and **will appear in the published article** if the submission is accepted. Please make sure it is accurate.<br><br>**Unfunded studies**<br>Enter: *The author(s) received no specific funding for this work.*<br><br>**Funded studies**<br>Enter a statement with the following details:<br>• Initials of the authors who received each award<br>• Grant numbers awarded to each author<br>• The full name of each funder<br>• URL of each funder website<br>• Did the sponsors or funders play any role in the study design, data collection and analysis, decision to publish, or preparation of the manuscript?<br>• **NO** - Include this sentence at the end of your statement: *The funders had no role in study design, data collection and analysis, decision to publish, or preparation of the manuscript.*<br>• **YES** - Specify the role(s) played.<br><br>* typeset	
**Competing Interests**<br><br>Use the instructions below to enter a competing interest statement for this submission. On behalf of all authors, disclose any competing interests that could be perceived to bias this work—acknowledging all financial support and any other relevant financial or non-financial competing interests.<br><br>This statement **will appear in the published article** if the submission is accepted. Please make sure it is accurate. View published research articles from *PLOS ONE* for specific examples.	The authors have declared that no competing interests exist.

**NO authors have competing interests**<br><br>Enter: *The authors have declared that no competing interests exist.*<br><br>**Authors with competing interests**<br><br>Enter competing interest details beginning with this statement:<br><br>*I have read the journal's policy and the authors of this manuscript have the following competing interests: [insert competing interests here]*<br><br>* typeset	
**Ethics Statement**<br><br>Enter an ethics statement for this submission. This statement is required if the study involved:<br><br>• Human participants<br>• Human specimens or tissue<br>• Vertebrate animals or cephalopods<br>• Vertebrate embryos or tissues<br>• Field research<br><br>Write "N/A" if the submission does not require an ethics statement.<br><br>General guidance is provided below. Consult the submission guidelines for detailed instructions. **Make sure that all information entered here is included in the Methods section of the manuscript.**	N/A

**Format for specific study types**<br><br>**Human Subject Research (involving human participants and/or tissue)**<br>• Give the name of the institutional review board or ethics committee that approved the study<br>• Include the approval number and/or a statement indicating approval of this research<br>• Indicate the form of consent obtained (written/oral) or the reason that consent was not obtained (e.g. the data were analyzed anonymously)<br><br>**Animal Research (involving vertebrate animals, embryos or tissues)**<br>• Provide the name of the Institutional Animal Care and Use Committee (IACUC) or other relevant ethics board that reviewed the study protocol, and indicate whether they approved this research or granted a formal waiver of ethical approval<br>• Include an approval number if one was obtained<br>• If the study involved *non-human primates*, add *additional details* about animal welfare and steps taken to ameliorate suffering<br>• If anesthesia, euthanasia, or any kind of animal sacrifice is part of the study, include briefly which substances and/or methods were applied<br><br>**Field Research**<br><br>Include the following details if this study involves the collection of plant, animal, or other materials from a natural setting:<br>• Field permit number<br>• Name of the institution or relevant body that granted permission	
**Data Availability**<br><br>Authors are required to make all data underlying the findings described fully available, without restriction, and from the time of publication. PLOS allows rare exceptions to address legal and ethical concerns. See the PLOS Data Policy and FAQ for detailed information.	Yes - all data are fully available without restriction

A Data Availability Statement describing where the data can be found is required at submission. Your answers to this question constitute the Data Availability Statement and **will be published in the article**, if accepted.<br><br>**Important:** Stating 'data available on request from the author' is not sufficient. If your data are only available upon request, select 'No' for the first question and explain your exceptional situation in the text box.<br><br>Do the authors confirm that all data underlying the findings described in their manuscript are fully available without restriction?	
**Describe where the data may be found in full sentences. If you are copying our sample text, replace any instances of XXX with the appropriate details.**<br><br>• If the data are **held or will be held in a public repository**, include URLs, accession numbers or DOIs. If this information will only be available after acceptance, indicate this by ticking the box below. For example: *All XXX files are available from the XXX database (accession number(s) XXX, XXX.).*<br>• If the data are all contained **within the manuscript and/or Supporting Information files**, enter the following: *All relevant data are within the manuscript and its Supporting Information files.*<br>• If neither of these applies but you are able to provide **details of access elsewhere**, with or without limitations, please do so. For example:<br><br>*Data cannot be shared publicly because of [XXX]. Data are available from the XXX Institutional Data Access / Ethics Committee (contact via XXX) for researchers who meet the criteria for access to confidential data.*<br><br>*The data underlying the results presented in the study are available from (include the name of the third party*	All relevant data are within the manuscript and its Supporting Information files.

and contact information or URL).<br>• This text is appropriate if the data are owned by a third party and authors do not have permission to share the data.<br><br>* typeset	
Additional data availability information:	Tick here if the URLs/accession numbers/DOIs will be available only after acceptance of the manuscript for publication so that we can ensure their inclusion before publication.

Manuscript

Click here to access/download;Manuscript;renamed_dc481.docx

Human encroachment, climate change and the loss of our archaeological organic cultural heritage: Accelerated bone deterioration on Ageröd, a revisited Scandinavian Mesolithic key-site in despair

Adam Boethius*[a]; Mathilda Kjällquist[b]; Ola Magnell[b]; Jan Apel[c]

*Corresponding author: adam.boethius@ark.lu.se

[a] Department of Archaeology and Ancient History, Lund University, Helgonavägen 3, 223 62 Lund, Sweden

[b] The Archaeologists, National Historical Museums, Sweden

[c] Department of Archaeology and Classical Studies, Stockholm University

Abstract

Ancient organic remains are essential for the reconstruction of past human lifeways and environments but are only preserved under particular conditions. Recent findings indicate that such conditions are becoming rarer and that ~~old~~ archaeological sites~~,~~ with previous good preservation, are losing their preservative qualities. To investigate this, we returned to the well-known Swedish Mesolithic site Ageröd I. Here we present the result of the re-excavation and the osteological analyses of the bone remains from the 1940s, 1970s and 2019 excavation campaigns of the site, to document and quantify changes in bone preservation and relate them to variations in soil conditions and on-site topography. The results indicate that the bone material has suffered from accelerated deterioration during the last 75 years. In some areas, this has led to a complete destruction of the remains while other areas, while suffering badly, are still able to preserve archaeological bones. We conclude that while Ageröd can still be considered an important site, it has lost much of the properties that made it unique and if no actions are taken to secure its well-being it will soon lose the organic remains that before modern encroachment and climate change had been pristinely preserved at the site for 9000 years. Finally, because nothing special, compared to most other archaeological sites, has happened to Ageröd during

these last 75 years, our results also raise questions of the state of organic preservation in other areas and call for a broad examination of our most vulnerable hidden archaeological remains.

Introduction

Researchers working with previously excavated archaeological sites have noticed that prehistoric organic remains are more rarely recovered today ~~if~~ compared to some decades ago, and that bones from earlier excavations are generally better preserved. This is a ~~silent and well-~~spread knowledge but has not been properly analysed, reported or measured. As a first step to remedy this, we conducted a small scale excavation campaign at the Middle Mesolithic site Ageröd I:HC, located in central Scania southern Sweden, in May 2019. Here, the faunal remains are analysed and compared with the remains recovered on previous excavation campaigns at the site, to measure the rate of deterioration that has occurred over the last 75 years.

There are few published reports concerning the ongoing soil acidification and its effect on non-excavated organic components on archaeological sites. However, in Sweden, around three decades ago concerns were raised regarding the effect of modern pollution on other types of cultural heritage remains. As a consequence of these concerns, the Swedish National Heritage Board initiated a project in 1988 to investigate how air pollution affected ancient monuments and buildings (Lindborg, 1990; Nord and Tronner, 1991). Part of this investment was also diverted into investigating archaeological artefact from earthbound contexts, focusing on metal objects. A correlation between modern soil acidification and accelerated metal corrosion during the last 50-100 years was demonstrated (Nord and Lagerlöf, 2002). Through EU financed research the investigations on archaeological materials in different soils could be expanded and also come to include bone deterioration and evaluations of different soils in selected European countries (Kars et al., 2002). The results indicated that the archaeological record was threatened and that in some areas organic material and non-noble metals faced destruction (Nord and Lagerlöf, 2002).

While indications of ongoing and threatened archaeological material was evident, limited follow-up investigations were ~~done~~. Also, most of the efforts from these studies focused on showing the present state of the archaeological objects and on differences in site-specific variations in bone degradation (e.g. Jans et al., 2002) or information on how to study and detect deterioration in different types of source materials (Huisman, 2009). Furthermore, site-specific deterioration in progress (i.e. comparison with museum collections) has been identified as an important aspect in furthering the information regarding the preservation of our ancient heritage (Nord et al., 2005). In 2011, Nicky Milner *et al* could show an alarming organic deterioration on site-specific level on the Mesolithic site Star Carr, by comparing excavated materials from different time periods (Boreham et al., 2011; Milner et al., 2011). The results from the Star Carr excavation, in combination with a growing body of evidence of accelerated organic degradation (Boreham et al., 2011; High et al., 2016; Hollesen et al., 2018; Hollesen and Matthiesen, 2015; Jans et al., 2002; Kars et al., 2002; Milner et al., 2011; Nord et al., 2005; Nord and Lagerlöf, 2002; Nord et al., 2007), suggest that more data on the status of ancient organic remains is needed. Furthermore, a general decline of wetlands (through drainage) in Europe is resulting in accelerated peat and gyttja degradation; which, because the anoxic peat layers in a natural unaltered peat bog have for millennia protected the archaeological materials (see e.g. Mathiessen and Jensen, 2004; Pedersen, 2014), calls for new investigations about the wellbeing of the organic parts of our common legacy and new means of quantifying contingent ongoing degradation of organic material at sites previously known for their good preservation.

The Ageröd site

Ageröd I is an Early Holocene site, Late Maglemose to Early Kongemose culture using south Scandinavian chronology (Sørensen, 2012), dated to roughly 8700-8200 cal BP. Ageröd I is located by a peat bog, which was once a shallow lake. The Ageröd peat bog is located the north of Lake Ringsjön in mid-Scania, southern Sweden (Fig. 1). This area is known for its rich Mesolithic sites with organic remains (Larsson, 1978a; 1983; Larsson et al., 1981 Reventlow, 1886; 1889; Sjöström, 1997;

Hammarstrand Dehman and Sjöström, 2009; Sjöström, 1995; Sjöström, 2004; 2013; Forssander, 1930; Reventlow, 1889; Rydbeck, 1930; Thomas, 1954).

Fig. 1. Map of Scandinavia zoomed in on the area of the Ageröd I site located on the ancient shore of the former shallow lake. Figure by Arne Sjöström.

Ageröd I was discovered in the 1930s (Forssander, 1938). However, due to the Second World War, it took until 1946-1949 until Carl Axel Althin at Lund University Historical Museum came to excavate the site. During the following ambitious, detailed and extensive excavations the site was divided into five major sections: A, B, C, HC, and D (Fig. 2). Ageröd I consists of three settlements with section A, C and HC as representing one settlement and Ageröd I:B and Ageröd I:D as two others (Larsson, 1978a). Althin recovered numerous finds and it soon became apparent that they had encountered the first well-preserved Middle Mesolithic site in Sweden displaying phenomenal preservation and large quantities of both bone materials and flint (Althin, 1954).

Fig. 2. Ageröd I with the original trench from the 1940s displayed on the background of an aerial photography from 1957.

New excavations were carried out by Lars Larsson between 1972 and 1974. These investigations allowed Larsson to make assessments of settlements patterns, chronology, seasonality, population size and resource exploitation (Larsson, 1978a) and present an overview of the bone and antler artefacts from the site (Larsson, 1978b). Apart from the previous excavations on the site (Fig. 3), the Ageröd bog has also gone through extensive quaternary geological analyses to establish a thorough stratigraphy of the bog, create pollen analytical dating sequences and to map the formation of the bog and its ancient shorelines (Nilsson, 1964a; b; 1967).

Fig. 3. Photography's from the 1940s and the 1970s excavation campaigns a) Surveying Ageröd I field prior to 1940s excavation, note fenced-in drainage ditch already in place. b) 1940s excavation in Ageröd I:HC. c) 1940s trench profile with detailed separation of different layers. d) 1970s excavation in Ageröd I. e) 1940s excavations under a sunshade in Ageröd I. f) Zooarchaeologist Herved Berlin excavating in Ageröd I:HC in the 1940s. g). 1970s excavation in Ageröd I, h) 1970s area topography of Ageröd I with the ongoing excavation in the background. i) 1940s excavation campaign camp facility setup. Photography: 1940s photos © Lund University Historical Museum; 1970s photos © Lars Larsson.

Most of the organic finds from Ageröd I consist of bone and antler refuse. However, while osteological analyses were ~~done~~ on the bones recovered from the Ageröd I: B & D sections in the 70s-excavations and published as a summary in the appendix to Larsson´s dissertation (Lepiksaar, 1978), the majority of the bones (from the I:HC section) have remained unpublished. The bones from previous excavations of the site have now been analysed (I:HC) and re-analysed (I:B, D) and are briefly published here (Tab. 1) along with the zooarchaeological analysis from the 2019 excavation campaign. The material from previous excavations of Ageröd I (B, D & HC) amount to a total of 4180 bone fragments determined to species or family level. From the Ageröd I:HC section 299 bone or antler artefacts have also been identified (Larsson, 1978b), which combined with the 18 bone and antler artefacts from the I:B & D section (Larsson, 1978a) place the bone and antler assemblage from Ageröd I as one of the largest and best-preserved Middle Mesolithic assemblages in northern Europe. When also considering other organic remains, recovered on the early excavations of the site, such as worked wood, resin, pollen and hazelnuts etc., the vast potential of the organic material culture from the site is highlighted.

Today, the area around Ageröd has been turned into farmland. To make the bog arable, farmers have, since the beginning of the 20th century, been digging ditches around and straight through the bog for drainage (Fig. 3a). It is safe to assume that the Ageröd bog started drying up with the digging of the initial drainage ditches. Indeed, compaction and degradation of the peat layers were, already in the 70s, noted when observations of a diminished peat thickness (more than 10 cm) were made when comparing the documentation of the peat layers from the excavations in 1948 with those done in 1973 (Larsson, 1978a:152).

The location of the Ageröd I site is fairly secluded and no roads, railroads or even modern houses have been constructed near the site. Consequently, the major disturbances to the sites come from background pollution (acidic precipitation, airborne exhaust etc.), climate change (larger fluctuation in groundwater levels due to warmer summers), the ditches draining the bog, ploughs turning the upper peat layers and previous excavations at the site.

The Excavatio[illegible]

Ageröd was revisited between the 16th and 24th of May 2019 with the purpose of investigating the *in situ* status of the organic material at the site. Five test pits were excavated in the close vicinity of the previous excavations at Ageröd I:HC (Fig. 4). The excavation was done by Lund University and The Archaeologists, National Historical Museums in Sweden. During the excavation campaign, 22 different researchers and archaeologists from 13 research institutes and universities visited and participated.

The test pits were located close to areas where most organic remains had been recovered during previous excavations. The absolute location of the original trenches was determined by using rectified aerial photographs from 1957, where the trenches could be seen (Fig. 2). This data was converted into the modern national grid system and tested for accuracy in the field by locating specific fix points hammered and drilled into large stones at the site on previous excavations, and by also bringing professor Larsson on to the site to discuss his fixpoints in the 1970s.

To facilitate interpretations, the Ageröd I:HC area was also divided into four zones. This included two zones designed to cover the area to the north of the added soil bank, zone 1 with original X-values between -9 to -14 and zone 2 encompassing the area of the added soil heap, with original x-values between -6 to -8. Two zones were also created to encompass the area on the lakeside of the ditch, zone 3 encompasses the first “shallow” parts of the former lake, with original x-values between -5 to -0, zone 4 encompasses the “deep” parts of the former lake including only positive x-values (+0 to +50).

Fig. 4. The square meter trenches from 2019 in relation to the former excavation trenches, the ditch draining the bog and the soil bank from when the ditch was dug and maintained throughout the last decades. The zone division was arbitrary selected to study differences in bone preservation between the driest (zone 1) and the wettest (zone 4) conditions.

Osteological analyses on the original osseous material, related to the rectified GIS-data, enabled quantifications of the number of bones found in each square meter on previous excavations. This

ensured that the new test pits could be placed in the areas where the best-preserved and largest quantities of bones had been recovered on previous excavations.

During the excavation five 1x1 meter trenches were excavated. In four of the five trenches, the archaeological layers were intact. In one case the new trench (259) was located partly within a previously unpublished and unknown trench. Consequently, the results from this trench is biased and excluded in the following discussions. The trenches were excavated by layer, and all layers (Fig. 5) were sampled for soil analyses and macrofossils. The results from the soil and macrofossil analyses will be published separately, together with histological analyses (Boethius et al., in prep). To ensure maximum possible recovery and to avoid missing small fragments, two-step water sieving (5mm mesh placed on top of a 2,5 mm mesh) was used. All soil from the major cultural layers in trench 201 and 217 were water sieved while the soil from the more peripheral layers were sampled and between 15-35 % of their soil was water sieved (Tab. Supplementary (S)1).

Fig. 5. Profiles from the four undisturbed trenches excavated in 2019. Blue arrow shows added soil from the drainage ditch, red arrow shows the upper peat layer, white arrow shows the cultural (white) layer and green arrow show the lower peat layer. The different layers are most evident in trench 201, in trench 205 the lower peat layer is almost completely gone and difficult to detect as it has deteriorated and become merged with the cultural layer. Trench 217 has the most amounts of added soil from the drainage ditch on top of the originally deposited layers and the ditch has been dug down into the moraine here as shown with the orange morainic soil in the added soil layer. Photography: Mathilda Kjällquist.

Methods

Zooarchaeological analysis

Renewed analyses of the osseous remains from previous excavations made it possible to quantify the material by studying species distribution, using Number of Identifiable Specimens (NISP), and to study bone preservation through weathering analyses. The bones from both old and new excavations were determined to species or family level using the reference collections at the Zoological museum and department of Archaeology and Ancient History at Lund University and the collections at the National Historical Museums in Sweden. The weathering analyses were done according to following criteria's:

Category 1: Light beige to medium brown, hard and shining surface no cracks, heavy bones, equivalent with Behrensmeyer (1978) stage 0. Category 2: Reddish, dark brown to black-brown, hard and shining surface, no or occasional cracks, heavy bones, Behrensmeyer stage 0-1. Category 3: Light to medium brown, dull, corroded surface, longitudinal cracks, exposure of cancellous bones, light bones. Behrensmeyer stage 1-2. Category 4: Light brown, dull surface, the outer surface is missing due to exfoliation, light bones. Behrensmeyer stage 3-5 (Fig. 6).

Fig. 6. Weathering I categories exemplified with bones from the 1940s excavation campaign. a: Wild boar calcaneus weathering category 1, b: Red deer astragalus weathering category 2, c: Red deer astragalus weathering category 3, d: Red deer astragalus weathering category 4 (lower end of category 4 spectrum), e: Red deer astragalus weathering category 4 (upper end of category 4 spectrum). Photography: Ola Magnell.

For weathering analyses on the bones from the new excavation, this was deemed inadequate, due to more extensive bone loss noted on the newly excavated bones (c.f. also the range of category 4 spectrum, Fig. 6 d & e). Consequently, prior to analyses, an additional weathering class was created (called weathering II) as a way of describing the details of the original weathering 4 category. Weathering II is, consequently, a four new categories detailed description of the original weathering I category 4, as follows: Category 5: all sides outer surface erosion less than 50%. Category 6: all sides outer surface erosion more than 50%. Category 7: no remaining surface, all sides average bone loss less than 5 mm. Category 8: no remaining surface, all sides average bone loss more than 5 mm (Fig. S1). For illustrative purposes, a Category 9 was also included. This is not an actual weathering category but an illustration to show a complete lack of unburnt bone in a particular trench. Because the bones from the old excavation campaigns were analysed using only 4 weathering categories, comparisons between new and old excavations were done using the first four weathering categories. For intra-excavation comparison, i.e. comparison between the different 2019 trenches, all weathering categories were used.

Material

This study focuses mainly on the osteological material from three separate excavations of Ageröd I. The material from the 1940s and 1970s excavations of Ageröd I (B, D & HC) amount to a total of 4180 bone fragments determined to species or family level. From the Ageröd I:HC section 299 bone or antler artefacts have also been identified (Larsson, 1978b), which combined with the 18 bone and antler artefacts from the I:B & D section (Larsson, 1978a) place the osseous remains from Ageröd I as one of the largest and best-preserved Middle Mesolithic assemblages in northern Europe. When also considering other organic remains, recovered on the early excavations of the site, such as worked wood, resin, pollen and hazelnuts etc., the vast potential of the organic material culture from the site is highlighted and constitute one of the reasons for why this site was selected for re-excavation and analysis.

During the 2019 excavation an additional 105 bone fragments, including 7 bone, tooth or antler artefact, were also recovered. Of these bone pieces, 61 fragments could be determined to species or family level, which means that the osseous material from Ageröd I encompass a total of 4241 determined bones (Tab. 1). In addition to the osseous remains the 2019 excavation revealed additional hazelnut shells and a variety of seeds from different plants, which were recovered by flotation of soil samples from each trench (Boethius et al. in prep). Furthermore, two pieces of resin were also recovered on the excavation. One of the resin pieces was found inserted in a slotted bone point (Fig. S2) and the other one was found with a flint microlith in its middle and bone dust remains on the outside, which suggests that this resin and flint piece had also previously been inserted in a slotted bone point (Fig. S3).

For comparison in this paper, only the determined bones from the Ageröd I:HC excavations have been used, which amounts to 3716 bone fragments from the old excavations and 61 fragments from the 2019 excavation. For the weathering analyses, 52 bones from the new excavations were used and 3392 from the old. All bones from the former excavations could not be assigned to an exact location. This is

both because of how the old excavations were conducted, where some of the areas of the site were crudely excavated and disregarded stratigraphic information, and/or because metadata, which could have connected specific bones to specific contexts/areas, have been lost in the years since the excavations. Consequently, not all bones from the old excavations could be included when analysing the preservation condition stratigraphically or spatially. In total, 181 bones from the 1970s zone 2, 519 bones from 1940s zone 1, 553 bones from 1940s zone 2, 872 bones from 1940s zone 3 and 358 bones from zone 4 were included.

Result

Abundance of species

Zooarchaeological analyses included recovered bone material from both the 1940s and the 1970s excavations of Ageröd I. The osteological material from the old excavations are extensive and yielded an array of different species, ~~though highly~~ dominated by ungulates. Because the new excavations were limited and only five square meters were excavated, the number of comparable bones are limited. When comparing the species distribution between the old and new excavations the results are, however, comparable in some aspects while they differ more in other (Tab. 1).

Tab. 1. Animal species distribution from all excavation campaigns at Ageröd I

Family	Species	Ageröd I:HC 1940s	Ageröd I:HC 1970s	Ageröd I:HC 2019	Ageröd I:HC Combined	Ageröd I:B 1940+1970s	Ageröd I:D 1940+1970s	Ageröd I Total
**Mammals (Mammalia)**								
Cervidae	Red deer (*Cervus elaphus*)	1426	62	22	1510	72	123	1705
	Roe deer (*Capreolus capreolus*)	326	20	7	353	18	12	383
	Elk (*Alces alces*)	219	20	5	244	10	11	265
	Cervidae indet.	79	1	3	83	42	3	128
Bovidae	Aurochs (*Bos primigenius*)	265	28	2	295	5	17	317
	*Aurochs (*Bos primigenius*)/ European bison (*Bison bonasus*)	51			51	1		52
	Aurochs (*Bos primigenius*)/ Cattle (*Bos taurus)*	9			9			9
	Cattle (*Bos taurus)*	1			1			1
Suidae	Wild boar (*Sus scrofa*)	848	64	12	924	54	34	1012
Equidae	Horse (*Equus caballus)*	1			1	5		6
	Large ungulate	17		6	23			23
Phocidae	Grey seal (*Halichoerus grypus*)	2			2			2
	Harp seal (*Pagophilus groenlandica*)	1			1			1
	Phocidae indet.	5			5			5
Ursidae	Brown bear (*Ursus arctos*)	62	3	2	67	3	2	72
Canidae	Wolf (*Canis lupus*)	4			4			4
	Red fox (*Vulpes vulpes*)				0	2		2
	Dog (*Canis familiaris*)	40	1		41	9	18	68
	Canidae indet.	5			5	1	1	7
Mustelidae	Badger (*Meles meles*)	11	1		12	1	2	15
	Otter (*Lutra lutra*)	4			4			4
	Pine marten (*Martes martes*)	11			11		2	13
	European polecat (*Mustela putorius*)	1			1			1
	Badger (*Meles meles*) / Otter (Lutra lutra)	1			1			1
Felidae	Wild cat (*Felis silvestris*)	3			3		1	4
	European lynx (*Lynx lynx*)	1			1			1
	Carnivora indet.	3		1	4	1		5
Erinaceidae	European hedgehog (*Erinaceus europaeus*)	2			2			2
Castoridae	Beaver (*Castor fiber*)	67	1	1	69	8	2	79
Sciuridae	Red squirrel (*Sciurus vulgaris*)	3			3			3
Hominidae	Human (*Homo sapiens*)	7			7			7
NISP	**Sum of identified mammals**	3475	201	61	3737	232	228	4197
**Birds (Aves)**								
Anatidae	Northern shoveler (*Anas clypeata)*	2			2			2
	Eurasian wigeon (*Anas penelope)*	1			1			1
	Northern pintail (Anas acuta)	2			2			2
	Red-breasted merganser *(Mergus serrator*)	1			1			1
	Bean goose (*Anser fabalis)*	1			1			1
	Brent goose/Barnacle goose (Branta bernicla/Branta leucopsis)	1			1			1
	Common goldeneye/tufted duck (Bucephala clangula/Aythya fuligula)	1			1			1
	Barnacle -/Greater whitefronted goose (Branta leucopsis/Anser albifrons)	1			1			1
	Anserini indet.	3			3			3
	Anatidae indet.	3			3			3
	Cygnus sp.	3			3			3
Podicipedidae	Great crested grebe (*Podiceps cristatus*)	3			3			3
	Grebe indet. (Podiceps sp.)	1			1			1
Rallidae	Common moorhen (*Gallinula chloropus*)	1			1			1
Phasianidae	Western capercaillie (*Tetrao urogallus)*	5			5			5
	Hen harrier (*Circus cyaneus*)	1			1			1
	White-tailed eagle (*Haliaeetus albicilla)*				0		1	1
	Aves indet.	2			2			2
NISP	**Sum of bird specimens**	32		0	32	0	1	33
**Fish (Pisces)**								
Cyprinidae	Tench *(Tinca tinca)*				0		1	1
Esocidae	Pike (Esox lucius)		1		1	1	1	3
NISP	**Sum of identified fish**	0	1	0	1	1	2	4
**Reptiles (Reptilia)**								
Emydidae	Emys orbicularis	6			6			6

In the HC sections, the biggest difference in species distribution between the old and the new excavation is the lack of smaller fur game animals, e.g. Canidae, Mustelidae and Felicidade. In the 2019 excavation bird bones are also completely lacking, which differs from previous excavations from which 32 bird bones representing a minimum of 13 species could be determined. Fish and reptile bones are also missing in the 2019 excavation, but they were rare in the former excavations as well. Regarding the fish, it is, however, difficult to know what the lack of fish bones from the former excavations indicate. No sieving was done on any of the former excavations and, given the difficulties in finding fishbones when not sieving with small mesh sizes (Boethius, 2016; Hultgreen et al., 1985; Segerberg, 1999), the lack of fish bones from the former excavations may indicate deficient recovery methods, as opposed to lack of fishing activities. Given the location of Ageröd I (on the shore of an ancient lake, Fig. 1) and a perceived general fish dependency in Southern Scandinavia at the time (Boethius, 2018a; Boethius & Ahlström, 2018) ~~a lack of fishing activities are unlikely~~. The species distribution from ungulates are more similar between the different excavation campaigns and only small differences can be observed, indicating that the deterioration had not affected the species abundance of larger mammals (Fig. 7).

*Fig. 7. Species distribution among the ungulates from the different excavation campaigns. *No specimens belonging to European bison have been determined, whereby it has been assumed that all larger bovid fragments belong to aurochs.*

Weathering

All appropriate bones (teeth and small bone fragments excluded) from the new and old excavation were subjected to a weathering analysis. Only bones from animal species found on both old and new excavations were compared species by species. Weathering comparisons were also made including all weathering-determinable bone fragments (Fig. 8).

Fig. 8. Mean weathering stages for the bones found during the 1940s, 1970s and the 2019 excavation campaigns, divided into the comparable species and a total for all determinable bone fragments combined. The severity of degradation increase with higher weathering degree numbers. Weathering degrees <3 are considered low, 3 medium and 4 high.

The results from the weathering studies give a clear indication that the osseous material has suffered increased diagenesis during the last 70 years. Furthermore, it is also apparent that the accelerated deterioration had started already in the 1970s. Consequently, the osseous remains had gone from a weathering average of around 2,8 in the 1940s (hard and heavy bones with occasional cracks) to 3,4 in the 1970s (lighter bones with larger cracks and exposure of cancellous bone) to in 2019 an average of 3,7 (light and heavily eroded bones, with loss of outer surface). There are some overlaps in bone preservation between the different excavations, i.e. the worst preserved bones from the 1940s are roughly comparable to the best-preserved bones from the 1970s and the worst bones from the 1970s are comparable to the best-preserved bones from 2019. However, the general trends clearly show a highly accelerated deterioration. When visually comparing the bones from the different decades of excavation (Fig. 9), the differences in preservation are also obvious.

Fig. 9. Organic bone preservation at Ageröd. a-d bones from 2019; e-i from old excavations. a) astragalus and calcaneus from wild boar found articulated in the transition between white layer and lower peat in trench 205, likely deposited in wet conditions with tendons and ligament still connected, weathering category 8. b) metatarsal from aurochs found in cultural/white layer in trench 217, weathering category 6. c) radius diaphysis from elk found in cultural /white layer in trench 2019, one of the best-preserved bone fragments from the 2019 excavation, weathering category 3. d) tibia from red deer found in cultural/white layer of trench 205, weathering category 7. e) drilled and ornated cervid antler from the 1940s, weathering category3. f) "net sinker" made from burr of red deer antler, from the 1940s excavation, weathering category 2. g) scapula from red deer found in the white layer in the 1970s, weathering category 3. h) femur diaphysis from aurochs from the 1940s, weathering category 2. i) slotted bone point from the 1940s, in mint condition with resin and inserted microliths. Photography: Ola Magnell and Adam Boethius.

Many of the bones from the 2019 excavation are heavily weathered (Fig. 9 a, b, d) whereby category 4 was deemed insufficient to use and additional weathering categories was created to study the degradation in the different trenches (see methods). Through this approach, it was possible to

establish the average weathering degree on the bones from the different species and to create a 2019 trench-weathering-average (Fig. 10), and to study the bone preservation in the different soil layers of each trench (Fig. S4).

Fig. 10. Mean weathering II values for the ungulates and for all bones combined, divided into the five trenches from the 2019 excavation. The severity of degradation increase *with higher weathering degree numbers. Weathering degrees <3 are considered low, 3 medium and 4-8 high, category 9 indicate lack of preserved unburnt bones.*

The bones from trench 201 and 217, both in zone 2, appears to be affected by bone deterioration to a lesser degree than the bones from trench 205, in zone 3. Interestingly, the two trenches with the best preservation were both located in the soil bank in zone 2, where the soil from when the drainage ditch had been heaped when the bog was first drained in the early 1900s. Another interesting observation regards trench 205 and 209, both located on the “lakeside” of the drainage ditch with the lowermost parts of the trenches still in wet condition. Trench 209 is completely lacking weathering determinable bone fragments (although two burnt and fully calcinated bones were found here) while in 205 the weathering degree seems to increase in the deepest and wettest areas of the trench. This trend is not reflected in the bones from the former excavations. When applying the same zone division to the old excavations, where the Ageröd HC area is divided into four zones from the driest conditions in zone 1 to the wettest condition in zone 4 (see Fig. 4 for zones), the bone preservation was originally best in the wettest and deepest areas of the former trenches (Fig. 11 & Fig. S5).

The low numbers of unburnt bones in 2019 zone 3 (with a complete lack of small bones in 205 and no unburnt bones in trench 209) is not caused by any disturbances to the layers as indicated by the fact that the lithic material is present here in comparable numbers to the other 2019 trenches (Fig. S6 & S7).

Fig. 11. Comparison between the mean weathering stages on the different excavation campaigns by zone (see Fig. 2 for zone specifics). The severity of degradation increase *with higher weathering degree numbers. Weathering degrees <3 are considered low, 3 medium and 4 high.*

Discussion

Certain special areas have for thousands of years managed to withstand the passing of time to allow preservation of vulnerable organic remains. These organic archives are vastly important for our current knowledge of past human activities and cultures and, indeed, of the environment itself (Clark 1936; Boethius et al., 2020). The Mesolithic site Ageröd has been one such archive were near to optimal conditions have left a glimpse into the organic material humans used and interacted with, some nine millennia ago. During the last 75 years, this record has suffered from an accelerated deterioration, which threatens its future existence. Unfortunately, nothing "special" has happened to the Ageröd site during this period. In other words, there have been no major constructions in the close vicinity of the site, the area is rather secluded and even though the wetland has been drained it has been done so by low-technological methods, i.e. without using mechanical pumps etc. Consequently, the Ageröd site is not highly divergent from most other sites in Northern Europe who have previously shown excellent organic preservation of archaeological organic material.

The evidence presented here focus on the osseous remains from the site. By a standard zooarchaeological analysis, on both the material from the old excavations and the new, it is possible to detect certain trends. Lately, several Mesolithic sites from Northern Europe have been revisited both physically and by going through old documentation and remains (Apel and Storå, 2018; Boethius, 2018b; Boethius et al., 2017; Groß et al., 2019). While old excavations are often fraught with different types of problems, which makes certain aspects of the recovered remains difficult to compare, much information can still be obtained. If studying the differences in species distribution between the different excavations at Ageröd, it is e.g. striking that so few remains from all small-bodied species are

lacking in the new excavation, despite water sieving. This is not a coincidence. The best-preserved areas in 1940 have now become the worst preserved areas.

We set up two test trenches in zone 3 (where the bones from the 1940s excavation had been best preserved) and one of them had no unburnt bones preserved and the other trench showed extensive corrosion leading to massive bone loss on each of the large bones that were still preserved. Here, the bone loss was often more than 5 mm on all sides of the bone, implying that most bones with a cortical bone thickness of less than 1 cm would be completely deteriorated. Also, the corrosion and weathering on the bones increase with increasing trench depth, with the most damaged bones still buried in wet conditions. This requires additional analyses to detect if the added degradation (and a complete loss of unburnt bones in trench 209) depends on lager fluctuations in the water table or if they are caused by the water turning acidic.

One indication of the processes involved can be gained from studying the bone histology, where the presence of oxidised pyrite was detected in bones from the trenches dug in the 2019 excavation campaign, while the histologically analysed bones from the 1940s and 1970s excavations showed only non-oxidised pyrite (Boethius et al. in prep). Pyrite is iron sulphide and only form under anoxic conditions due to the action of sulphate-reducing bacteria. If oxygen is re-introduced into the environment, these pyrite grains will oxidize and turn into iron-hydroxides at the same time as sulphuric acid is produced (Hollund et al., 2012; Turner-Walker, 1999). The released sulphuric acid will etch and damage the bone, and cause pH levels to drop in the area. Indeed, the fact that oxidised pyrite was only detected in the bones from the 2019 excavations and the bones from previous excavations show only non-oxidised pyrite, suggests that the local soil environment has gone from stable anoxic to oxic conditions (Boethius et al. in prep).

Consequently, it is conceivable that the observed elevated bone destruction in the deepest and wettest parts of trench 205, and the complete bone destruction in trench 209, is caused by the groundwater having turned acidic by sulphuric acid seeping down from the cultural layers above.

Indeed, preliminary results from the excavation suggested a correlation between lower pH levels in the soil with increasing trench depths, when using a pH and moisture soil probe (EAN 2007005725967). These observations were also confirmed by the soil chemical analyses, where the two trenches without bone remains (209 and 259[1]) are the most oxygenised and have the lowest pH levels (between pH 4.2 and 5.0), thus displaying the worst soil properties for organic preservation (Boethius et al., in prep).

In trench 205, where bone preservation is worst among the areas where bones are still preserved, the soil analyses show an almost neutral pH-value. However, in trench 205 the lowermost soil sample was extracted between 15-18 cm from the underlying moraine (the moraine is roughly corresponding with the groundwater level) while the soil samples in 209 was extracted only 5 cm from the moraine and are thus directly connected to the groundwater. Furthermore, no bones were recovered from the lowermost 10 cm in trench 205 (where it was wet) and, just as in trench 209 and 259, the soil in 205 had oxidated (Boethius et al., in prep), suggesting that the soil has lost all its buffering qualities.

These observations are in agreement with the histological analyses. The microstructure of the interior central parts of the cortical bones from the wet conditions in trench 205 are among the histologically best-preserved bones from 2019 (Boethius et al., in prep), despite them suffering from elevated surface etching and corrosion. This shows that the inner cortical microstructure of the bones from the previous best-preserved area, zone 3, is still mostly unaffected by bioerosion and microbial activity (Boethius et al., in prep).

Surface corrosion is also observed on the palaeobotanical record from zone 3. On both water-pepper (*Polygonum hydropiper*) seeds and hazelnut (*Corylus avellana)* shells similar surface corrosion and etching, as detected on the bones in trench 205 (Figs. 9a, d), is noted (Fig. 12). Also, the palaeobotanical analyses show earthworm eggs (*Lumbricus sp.)* in the lower areas of both trench 201 and 209 (Boethius et al., in prep). Since earthworms cannot live in anoxic layers, this is further indication of oxygenated soil. More importantly, the inclusion of earthworms will also act to increase soil oxygenation, because

[1] However, as mentioned above, parts of trench 259 had been disturbed by a former non-registered trench and the results are, consequently, biased.

of how earthworms loosen up soil with their movements, which will cause further drainage and oxygen admittance, in a (from a perspective of archaeo-organic preservation) downward spiral.

Fig. 12. Seeds from water-pepper (a) and hazelnut (b) from trench 209 showing signs of corrosion and etching caused by an oxygenated and acidic environment. Photography: Santeri Vanhanen

The observed accelerated bone surface destruction in the formerly best-preserved areas of zone 3 and the observed corrosion on palaeobotanical remains from the same area is, given the above-mentioned observations, related to an increased groundwater acidity and fluctuations in the groundwater table. The acidity is likely caused by a combination of acid precipitation and re-oxygenated soils, which cause the pyrite to oxidise and add sulphuric acid to the local groundwater. Without a long-term monitoring program for the site, it is not possible to detect fluctuations in the groundwater level. However, the soil acidity observed in the lowermost areas of trench 209 (and the absence of unburnt bones), combined with the lack of bones in the lowermost 10 cm of trench 205 and the surface corrosion on the bones in the areas stratigraphically above these wet conditions, with increasing degradation with increasing recovery depth (Fig. S4), makes it plausible that the bone surface corrosion observed in 205 has been caused by fluctuation in the groundwater table. It is conceivable that water fluctuations have enabled acidic groundwater to corrode the bone surface during high groundwater levels, but for limited periods. If acidic groundwater, with pH levels matching those observed at the bottom of trench 209 (pH 5,2), had been in contact with bones recovered in 205 for extended periods the bones that were still preserved here would likely have dissolved. This is further strengthened by the lack of small unburnt bones in the trench and the high weathering levels on the remaining large bones, from which more than 5 mm of the outer bone surface has deteriorated.

Another interesting observation is that the best-preserved bones from 2019 were found in the layers under the soil-heap from the drainage ditch. While the ditch itself was a full-scale destruction of the archaeology in the area where the ditch was dug, it also appears as if the combination of drainage and

added soil seems to have acted to preserve the bone remains. The bones from the trenches situated in the soil bank are affected by an accelerated deterioration, but it is less severe here than in the other areas (Figs 11 & S5). The best-preserved bones from zone 2, under the soil bank, are comparable to the worst preserved bones from the 1970s, which suggest that the combination of a buffering soil on top of the cultural remains and the removal of what seems to be acidic groundwater, does act to delay bone degradation. However, the offered protection is not optimal and while it delays the deterioration, it does not protect the remains. Thereby, merely adding soil on top of threatened cultural remains will only offer a short-term "time-to-act" solution and not a long-term protection. Consequently, while the added soil has offered some buffer against degradation, time is running out. Oxygen has entered the cultural layers down into the deepest parts of the trenches, evident through both earthworm eggs, large cracks in the peat and soil (caused by the soil dehydration), and soil chemical analyses, which show that the soils are oxidised even in the deepest lying layers (Boethius et al., in prep). This is causing an accelerated humification and peat degradation process and a non-reversible deterioration of the archaeological organic remains, which, if not stopped, will cause the organic remains at Ageröd to disappear in a not too distant future.

Concluding remarks

The organic archaeological remains on Ageröd are threatened by an accelerating organic destruction. The best-preserved areas in the 1940s have now become the worst areas for organic preservation. This is related to an acidification of the groundwater in the bog, which might be related to both re-oxygenated soil conditions, causing the release of sulphuric acid, as well as acid precipitation. This raises serious questions about the preservation condition on other wetlands in Northern Europe with similar conditions.

If oxygen is introduced to anoxic layers, through drainage of wetlands, extreme drought leading to dry cracks in the previously wet peat, or indeed, previous archaeological excavations, it might start a reaction were pyrite oxidize and in the process develop sulphuric acid. If this should happen to layers

that will ones again become wet the sulphuric acid might be released into the surrounding water, causing pH-levels in the groundwater to drop. This can be connected to the differences in preservation observed at the different zones of Ageröd. The water in the wet areas might encompass and surround the bones with acidic groundwater, while the acidity released in the dry zones will seep further down in the ground, until reaching the groundwater and are thus adding further acidity to the affected basin while preventing the bones in the dry conditions from being "soaked" in acidic water.

~~Through our small-scale~~ excavation, we have highlighted the problems of organic deterioration~~, we~~ have shown that the bones are suffering badly from accelerated degradation and that in some areas this has destroyed all bone remains, which only 75 years ago had often been almost pristinely preserved for over 9000 years. The cause for alarm is great and it is likely that the bones even further out in the Ageröd basin has been even more affected, which raises questions of the well-being of the organic remains not only on Ageröd I but also on e.g. Ageröd V, which had remarkable organic preservation in the excavations in the late 1970s (Larsson, 1983). Further investigations are needed, on both the soil and the organic remains from Ageröd I, but also from other parts of the Ageröd basin.

Furthermore, we have to start investigating the preservation conditions at other sites to find out how modern encroachments, acidification and climate change have affected the organic remains elsewhere. For example, the drainage of wetlands has been widely practised throughout the world to create more arable land and/or improve wood production (cf. e.g. Armentano and Menges, 1986; Zedler, 2003)~~, which~~ have had dire consequences for the preservation of organic remains (Koivisto, 2017). Extreme weather situations, with summers of extreme drought (e.g. the summer of 2018), are likely also responsible for the drying of wetlands and lowering of the groundwater table. In addition, anthropogenically induced climate change does seem to increase the likelihood of extreme weather situations, e.g. both droughts and extreme rainfalls (National Academies of Sciences and Medicine, 2016). This, if considered in the light of recent studies suggesting that droughts are expected to drastically increase in the future (Grillakis, 2019), is cause for alarm concerning the future of ancient organic preservations in wetlands in general.

Our results from Ageröd I are alarming and calls for a large-scale examination of other archaeological sites with organic preservation. The hidden archaeological landscape needs to be re-examined, site-specific deteriorations must be quantified and local prerequisites must be evaluated before appropriate protective actions to secure our ancient heritage can be taken. The time to act is now and if we wait, we might be in a situation where no matter what actions we take, all organic remains at Ageröd (and possibly elsewhere) will be lost foreve[illegible].

Acknowledgement

We would like to thank Lars Larsson for helping us locate the old excavation trenches at Ageröd and for sharing his profound knowledge of the site. We would also like to thank Arne Sjöström and Santeri Vanhanen for letting us use their figures. Lastly, we are grateful for the assistance of our collaborators in the Stone Age for the Future Network and everybody else who volunteered and assisted during our excavation of the site.

Funding sources

We are grateful for the financial support from Crafoord foundation (nr. 20180631), Lennart J. Hägglunds foundation for archaeological research and education and the Swedish National Heritage Board (RAÄ-2018-3237) who have made this research possible.

References

Althin, C.-A. 1954. *The Chronology of the Stone Age Settlement of Scania, Sweden,* Acta Archaeologica Lundensia. Series in 4°, 1, Lund.

Apel, J. & Storå, J. 2018. The Pioneer settlements of Gotland—a behavioural ecology approach. *In:* Persson, P., Skar, B., Breivik, H. M., Riede, F. & Jonsson, L. (eds.) *The Ecology of Early Settlement in Northern Europe: Conditions for Subsistence and Survival.* 277-309. Sheffield: Equinox.

Armentano, T. & Menges, E. 1986. Patterns of change in the carbon balance of organic soil-wetlands of the temperate zone. *The Journal of Ecology*, 755-774.

Behrensmeyer, A. K. 1978. Taphonomic and ecologic information from bone weathering. *Paleobiology,* 4**,** 150-162.

Boethius, A. 2016. Something rotten in Scandinavia: The world's earliest evidence of fermentation. *Journal of Archaeological Science,* 66**,** 169-180.

Boethius, A. 2018a. *Fishing for ways to thrive: integrating zooarchaeology to understand subsistence strategies and their implications among Early and Middle Mesolithic southern Scandinavian foragers,* Acta Archaeologica Lundensia Series in 8°, vol. 70. Studies in Osteology 4*,* Lund: Lund University.

Boethius, A. 2018b. Huseby klev and the quest for pioneer subsistence strategies: Diversification of a maritime lifestyle. *In:* Persson, P., Skar, B., Breivik, H. M., Riede, F. & Jonsson, L. (eds.) *The Ecology of Early Settlement in Northern Europe - Conditions for Subsistence and Survival.* 99-128. Sheffield: Equinox.

Boethius, A. & Ahlström, T. 2018. Fish and resilience among Early Holocene foragers of southern Scandinavia: A fusion of stable isotopes and zooarchaeology through Bayesian mixing modelling. *Journal of Archaeological Science,* 93**,** 196-210.

Boethius, A., Bergsvik, K. A. & Nilsson, B. 2020. Knowledge from the ancient sea – a long-term perspective of human impact on aquatic life in Mesolithic Scandinavia. *The Holocene,* Online first. https://doi.org/10.1177/0959683619895585.

Boethius, A., Storå, J., Hongslo Vala, C. & Apel, J. 2017. The importance of freshwater fish in Early Holocene subsistence: exemplified with the human colonization of the island of Gotland in the Baltic basin. *Journal of Archaeological Science: Reports,* 13**,** 625–634.

Boethius, A., Hollund, H., Linderholm, J., Vanhanen, S., Kjällquist, M., Magnell, O., Apel, J., in prep. Quantifying archaeo-organic deterioration – a multiproxy approach to understand the accelerated deterioration of the ancient organic cultural heritage on the Swedish Mesolithic site Ageröd.

Boreham, S., Conneller, C., Milner, N., Taylor, B., Needham, A., Boreham, J. & Rolfe, C. J. 2011. Geochemical indicators of preservation status and site deterioration at Star Carr. *Journal of Archaeological Science,* 38, 2833-2857.

Forssander, J. 1930. En märklig stenåldersboplats vid Sjöholmen. Meddelanden från Lunds universitets historiska museum, IV, 25-42.

Forssander, J. 1938. Det äldsta Skåne. Skånes hembygdsförbunds årsbok. 1-14. Lund.

Grillakis, M. G. 2019. Increase in severe and extreme soil moisture droughts for Europe under climate change. *Science of The Total Environment,* 660, 1245-1255.

Groß, D., Lübke, H., Schmölcke, U. & Zanon, M. 2019. Early Mesolithic activities at ancient Lake Duvensee, northern Germany. *The Holocene,* 29, 197-208.

Hammarstrand Dehman, C. & Sjöström, A. 2009. *Mesolitiska lämningar i Rönneholms mosse. Arkeologisk förundersökning 2008. Hassle 32: 18, Stehag socken, Eslövs kommun, Skåne,* Lund: Institutionen för arkeologi och antikens historia, Lunds universitet,.

High, K., Milner, N., Panter, I., Demarchi, B. & Penkman, K. E. 2016. Lessons from Star Carr on the vulnerability of organic archaeological remains to environmental change. *Proceedings of the National Academy of Sciences*, 201609222.

Hollesen, J., Callanan, M., Dawson, T., Fenger-Nielsen, R., Friesen, T. M., Jensen, A. M., Markham, A., Martens, V. V., Pitulko, V. V. & Rockman, M. 2018. Climate change and the deteriorating archaeological and environmental archives of the Arctic. *antiquity,* 92, 573-586.

Hollesen, J. & Matthiesen, H. 2015. The influence of soil moisture, temperature and oxygen on the oxic decay of organic archaeological deposits. *Archaeometry,* 57, 362-377.

Hollund, H. I., Jans, M. M. E., Collins, M. J., Kars, H., Joosten, I. & Kars, S. M. 2012. What happened here? Bone histology as a tool in decoding the postmortem histories of archaeological bone from Castricum, The Netherlands. *International Journal of Osteoarchaeology,* 22, 537-548.

Huisman, D. J. (ed.) 2009. *Degradation of archaeological remains,* Den Haag: SDU Uitg.

Hultgreen, T., Johansen, O. & Lie, R. 1985. Stiurhelleren i Rana Dokumentasjon av korn, husdyr og sild i yngre steinalder. *Viking,* 48**,** 83-102.

Jans, M., Kars, H., Nielsen–Marsh, C., Smith, C., Nord, A., Arthur, P. & Earl, N. 2002. In situ preservation of archaeological bone: a histological study within a multidisciplinary approach. *Archaeometry,* 44**,** 343-352.

Kars, E., Kars, H., Arthur, P., Borg, G. C., Bruno, B., Collins, M., Christensson, E. & Earl, N. 2002. The degradation of bone as an indicator for the deterioration of the European archaeological property. *Kars, H. and Kars, E.(eds.)***,** 1-297.

Koivisto, S. 2017. Archaeology of Finnish wetlands: with special reference to studies of Stone Age stationary wooden fishing structures, Helsinki: Unigrafia.

Larsson, L. 1978a. *Ageröd I:B - Ageröd I:D: a study of early Atlantic settlement in Scania,* Acta Archaeological Lundensia Series in 4°, 12*,* Lund: Lund university.

Larsson, L. 1978b. Mesolithic antler and bone artefacts from Central Scania. *Meddelanden från Lunds universitets historiska museum,* 1977-1978**,** 28-67.

Larsson, L. 1983. *Ageröd V: An Atlantic bog site in central Scania,* Lund: Institute of Archaeology, University of Lund

Larsson, L., Meiklejohn, C. & Newell, R. R. 1981. Human skeletal material from the Mesolithic site of Ageröd I: HC, Scania, Southern Sweden. *Fornvännen,* 76**,** 161-167.

Lepiksaar, J. 1978. Bone remains from the mesolithic settlements Ageröd I:B and Ageröd I:D. *In:* Larsson, L. (ed.) *Ageröd I:B – Ageröd I:D, a study of early atlantic settlement in Scania.* 234-244. Lund: Lund university.

Lindborg, U. 1990. Luftföroreningar och kulturminnen. Handlingsplan 90. *Konserveringstekniska Studier.* 1-75. Stockholm: Riksantikvarieämbetet.

Mathiessen, H. & Jensen, P. 2004. Bevaring i Åmosen - hvor vådt er vådt nok. *In:* Fischer, A. & Pedersen, L. (eds.) *Kulturarv i Sjællands grønne hjerte.* 43-52. København.

Milner, N., Conneller, C., Elliott, B., Koon, H., Panter, I., Penkman, K., Taylor, B. & Taylor, M. 2011. From riches to rags: organic deterioration at Star Carr. *Journal of Archaeological Science,* 38**,** 2818-2832.

National Academies of Sciences, E. & Medicine 2016. Attribution of extreme weather events in the context of climate change. Washington, DC: National Academies Press.

Nilsson, T. 1964a. Entwicklungsgeschichtliche studien im Ageröds mosse, Schonen. *Lunds universitets årsskrift,* NF Avd. 2. Bd 59. Nr 8.

Nilsson, T. 1964b. Standardpollendiagramme und C14-Datierungen aus dem Ageröds Mosse im mittleren Schonen. *Lunds universitets årsskrift,* NF Avd. 2. Bd 59. Nr 7.

Nilsson, T. 1967. *Pollenanalytische Datierung mesolithischer Siedlungen im Randgebiet des Ageröds Mosse im mittleren Schonen,* Lund: Acta Universitatis Lundensis.

Nord, A. G., Kars, H., Ullén, I., Tronner, K. & Kars, E. 2005. Deterioration of archaeological bone—a statistical approach. *Journal of Nordic Archaeological Science,* 15**,** 77-86.

Nord, A. G. & Lagerlöf, A. 2002. *Påverkan på arkeologiskt material i jord: redovisning av två forskningsprojekt,* Stockholm: Riksantikvarieämbetets förl.

Nord, A. G. & Tronner, K. 1991. *Stone weathering – air pollution effects evidenced by chemical analysis,* Stockholm: Riksantikvarieämbetet.

Nord, A. G., Ullén, I. & Tronner, K. 2007. *" Bara naturlig försurning": Program för övervakning–en fallstudie över försurningens inverkan på fornlämningar,* Stockholm: Riksantikvarieämbetet.

Pedersen, K. B. 2014. On dating the late palaeolithic – a comment. *In:* Riede, F. & Tallavaara, M. (eds.) *Lateglacial and postglacial pioneers in northern Europe.* 23-33. Oxford.

Reventlow, C. D. 1886. Fynden från Ringsjön. *Kongliga vitterherhets historie och antiqvitets akademins månadsblad,* 1886**,** 140-144.

Reventlow, C. D. 1889. Fynden från Ringsjön. *Kongl. vitterherhets historie och antiqvitets akademins månadsblad,* 1889**,** 77-96.

Rydbeck, O. 1930. The earliest settling of man in Scandinavia. *Acta Archaeologica,* 1**,** 55-87.

Segerberg, A. 1999. *Bälinge mossar. Kustbor i Uppland under yngre stenåldern,* Aun 26*,* Uppsala: Department of Archaeology and Ancient History, Uppsala University.

Sjöström, A. 1995. *Grävningsrapport. Rönneholm 4, 5, 7 och 8. Arkeologisk förundersökning av fyra mesolitiska boplatser å Hassle 32: 18, Stehag socken, Eslövs kommun, Skåne,* Lund: Department of Archaeology and Ancient History, Lund University.

Sjöström, A. 1997. Ringsjöholm. A Boreal-Early Atlantic Settlement in Central Scania, Sweden. *Lund Archaeological Review,* 3**,** 5-20.

Sjöström, A. 2004. *Rönneholm 6-10, 12, 14 och 15: arkeologisk undersökning av ett mesolitiskt boplatskomplex i Rönneholms mosse. Hassle 32: 18, Stehag socken, Eslövs kommun, Skåne,* Lund: Department of Archaeology and Ancient History, Lund University.

Sjöström, A. 2013. *Mesolitiska lämningar i Rönneholms mosse. Arkeologisk förundersökning 2012. Hassle 32: 18, Stehag socken, Eslövs kommun, Skåne,* Lund: Department of Archaeology and Ancient History, Lund University.

Sørensen, M. 2012. The arrival and development of pressure blade technology in southern Scandinavia. *In:* Desrosiers, P. M. (ed.) *The Emergence of Pressure Blade Making. From Origin to Modern Experimentation.* 237-259. New York: Springer.

Turner-Walker, G. 1999. Pyrite and bone diagenesis in terrestrial sediments: Evidence from the West Runton freshwater bed. *Bulletin of the Geological Society of Norfolk***,** 3-26.

Thomas, S. E. 1954. Sjöholmen, Site 179. A Re-Examination. *In:* Althin, C.-A. (ed.) *The Chronology of the Stone Age Settlement of Scania, Sweden.* Acta Archaeologica Lundensia, Series in 4°, 1. Lund.

Zedler, J. B. 2003. Wetlands at your service: reducing impacts of agriculture at the watershed scale. Frontiers in Ecology and the Environment, 1, 65-72.

Figure

Click here to access/download;Figure;Fig. 1.tif

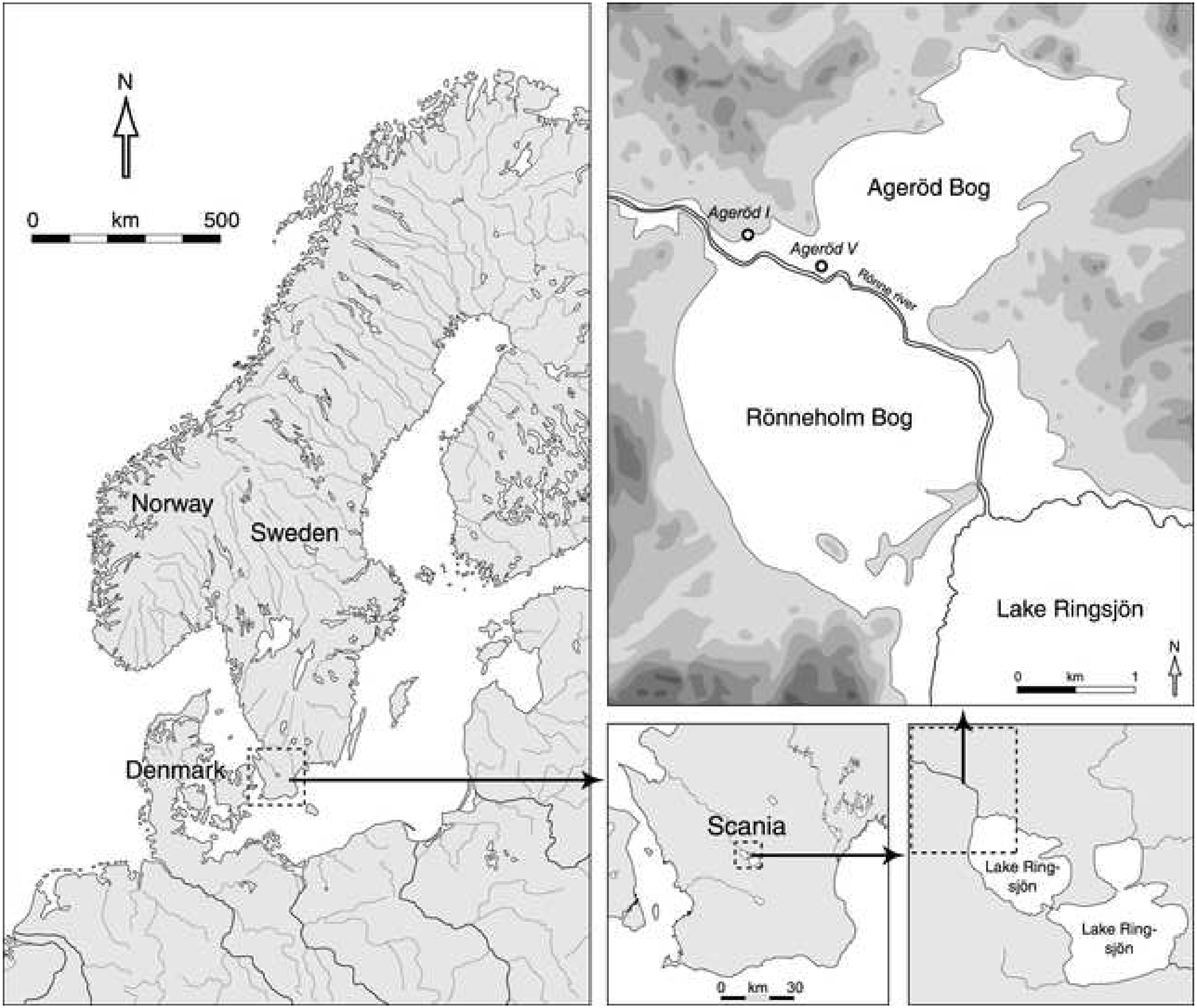

Figure

Click here to access/download;Figure;Fig. 2.tif

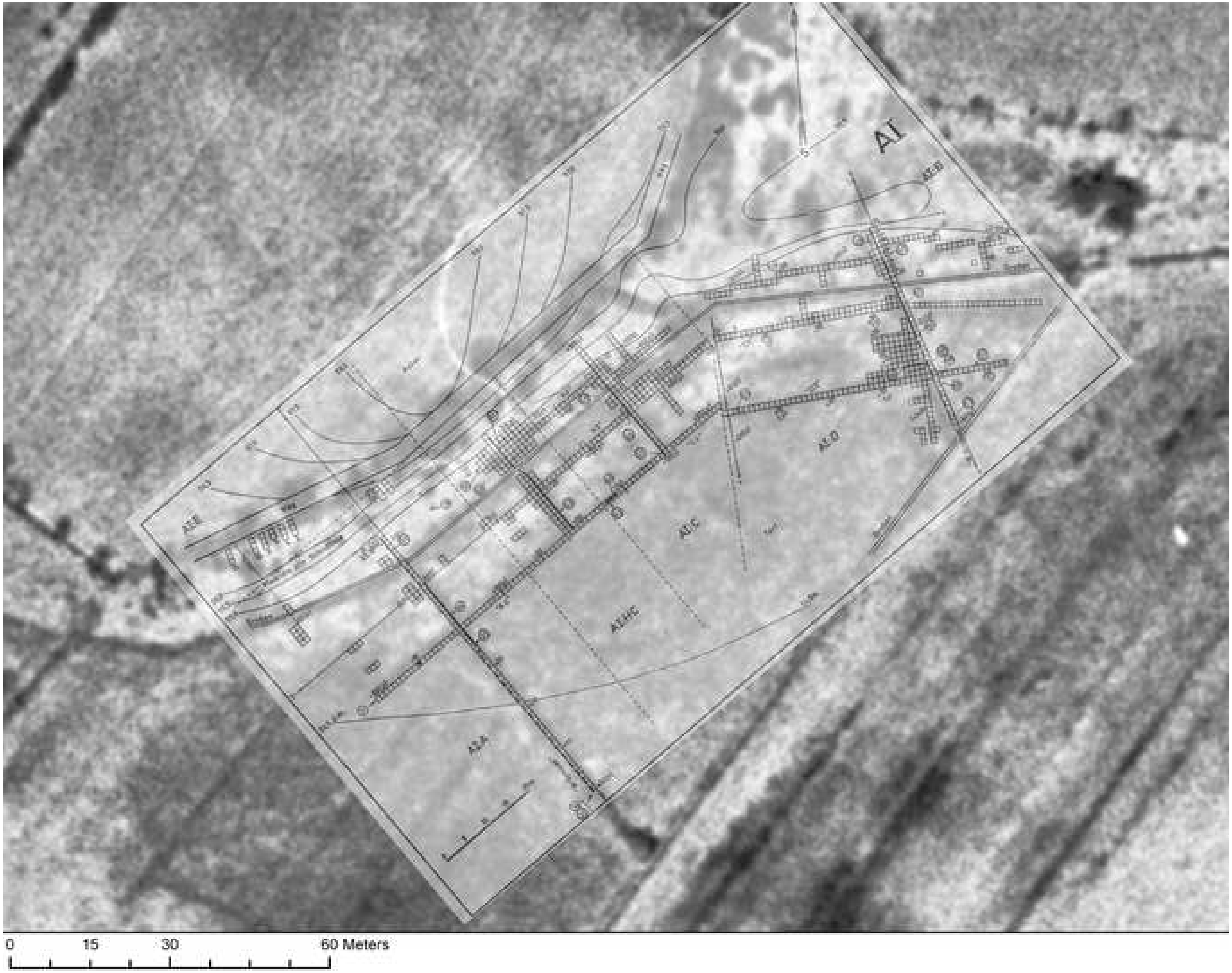

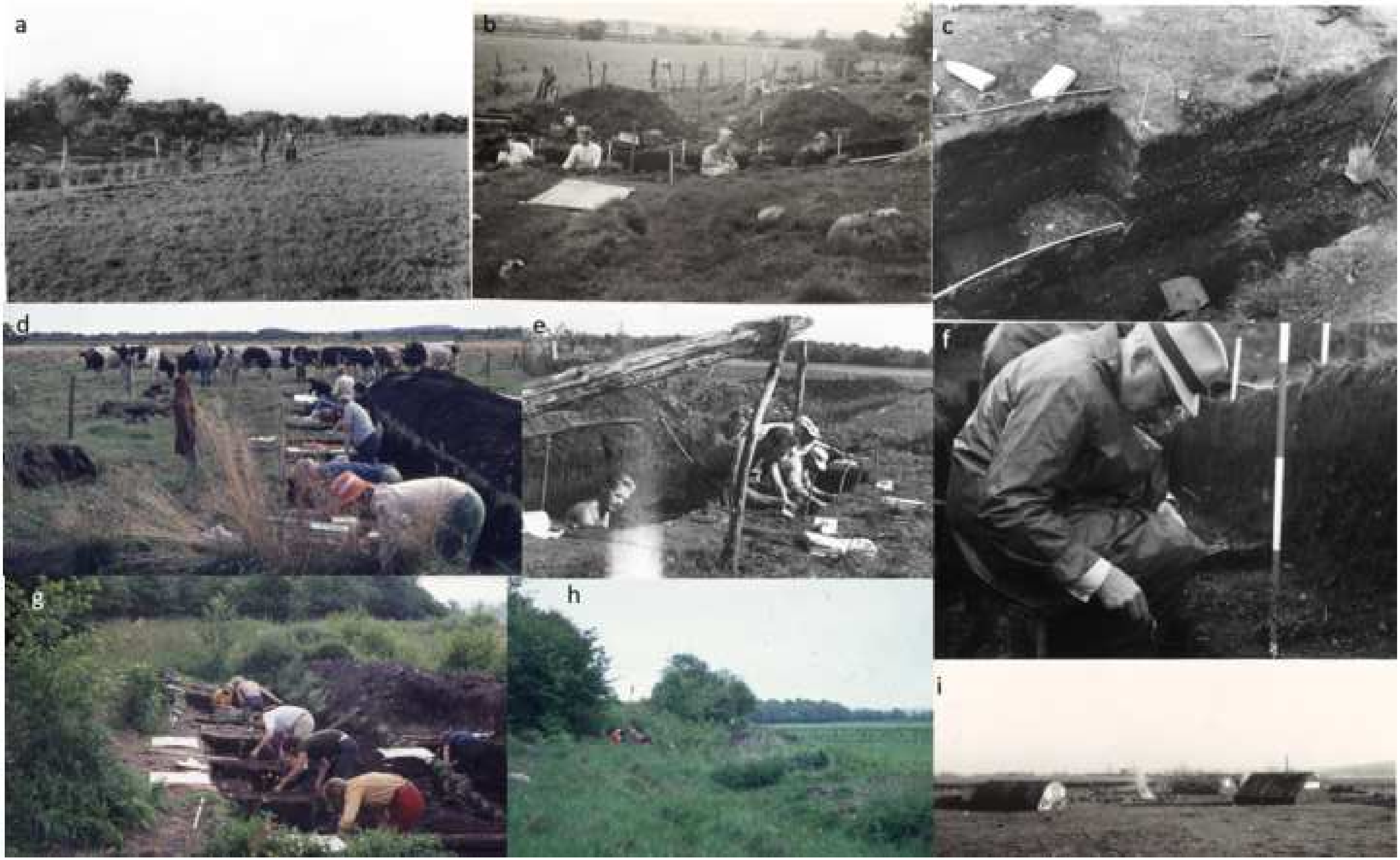

a
b
c
d
e
f
g
h
i

Figure

Click here to access/download;Figure;Fig. 4.tif

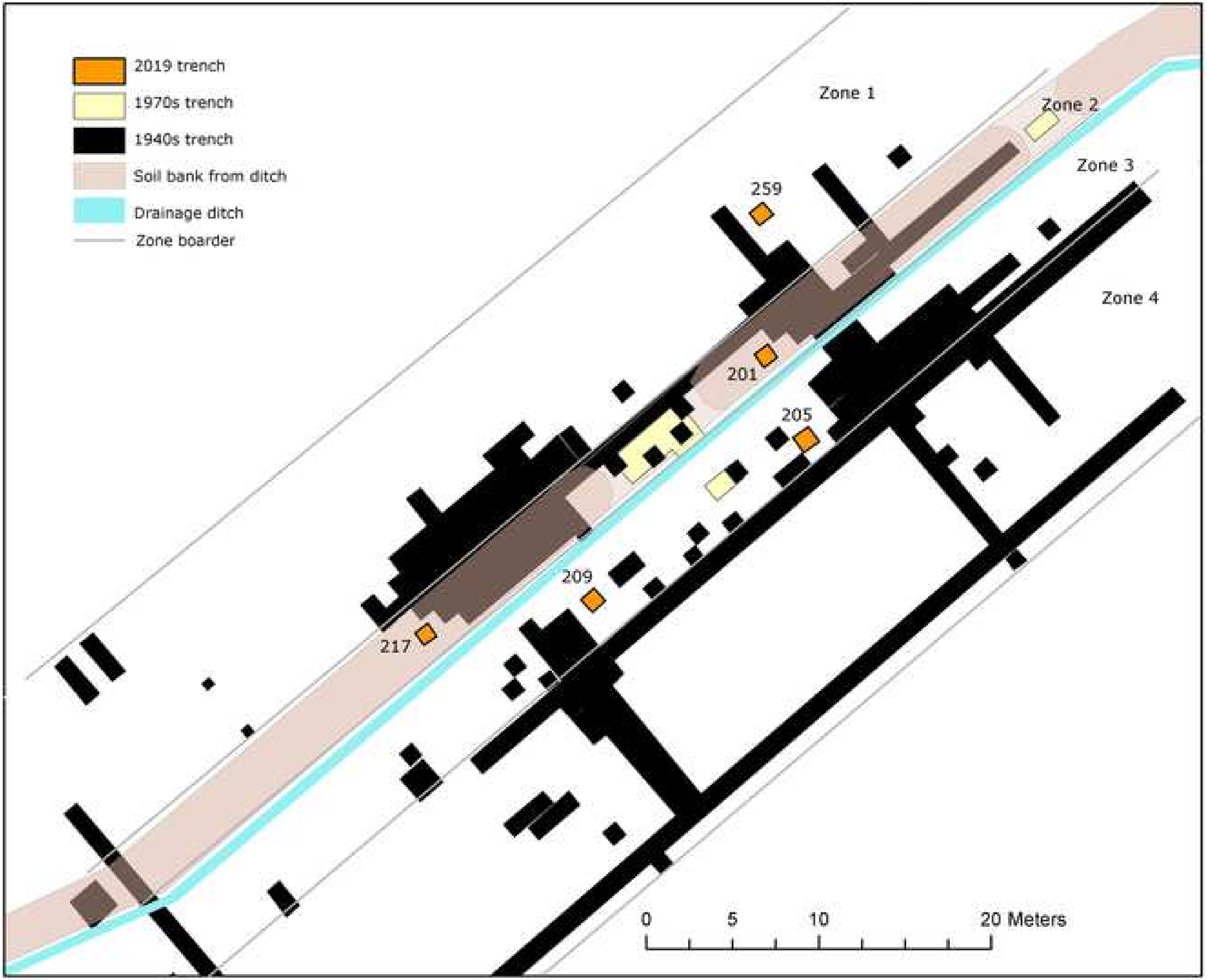

Click here to access/download;Figure;Fig. 5.tif

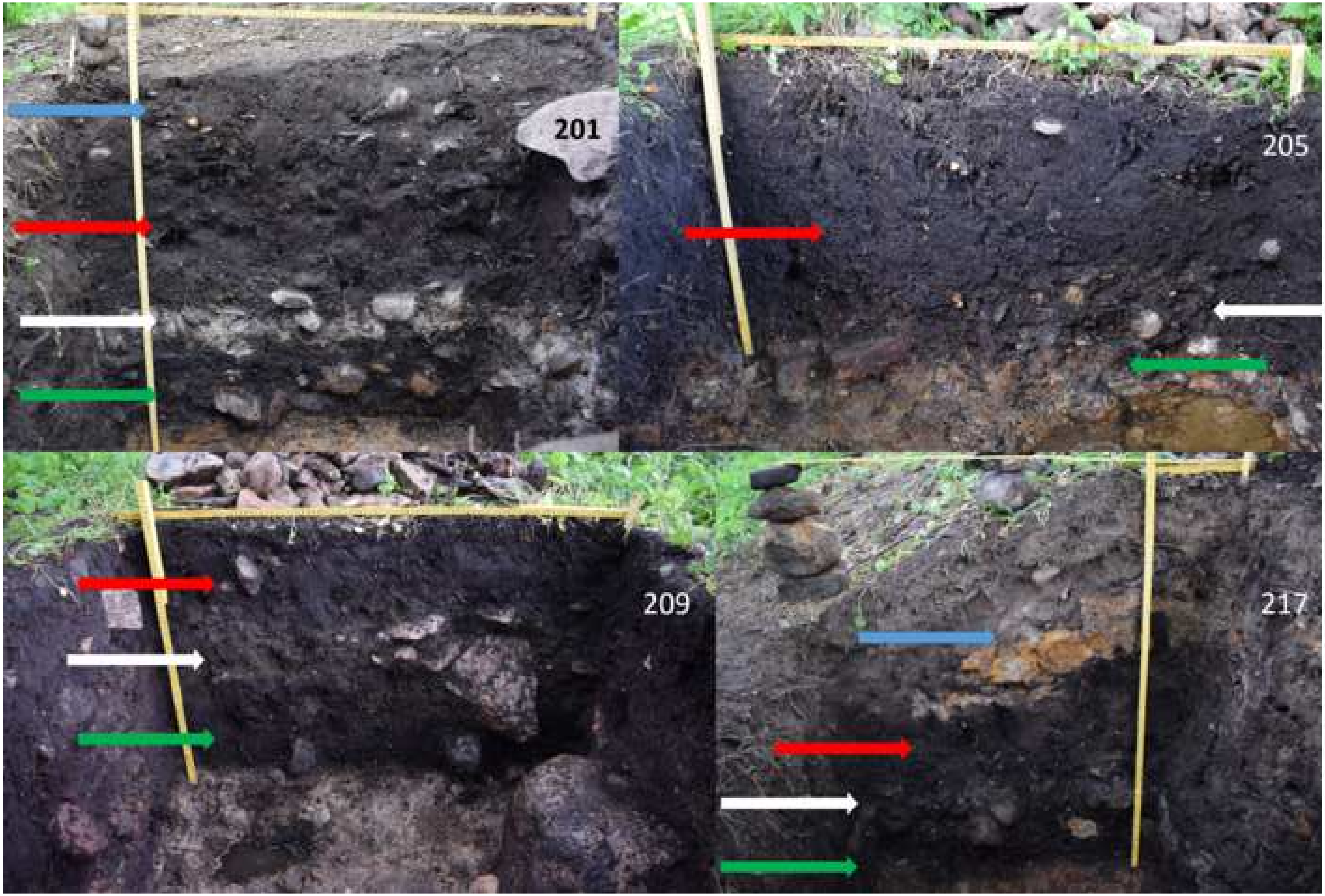

Figure

Click here to access/download;Figure;Fig. 6.tif

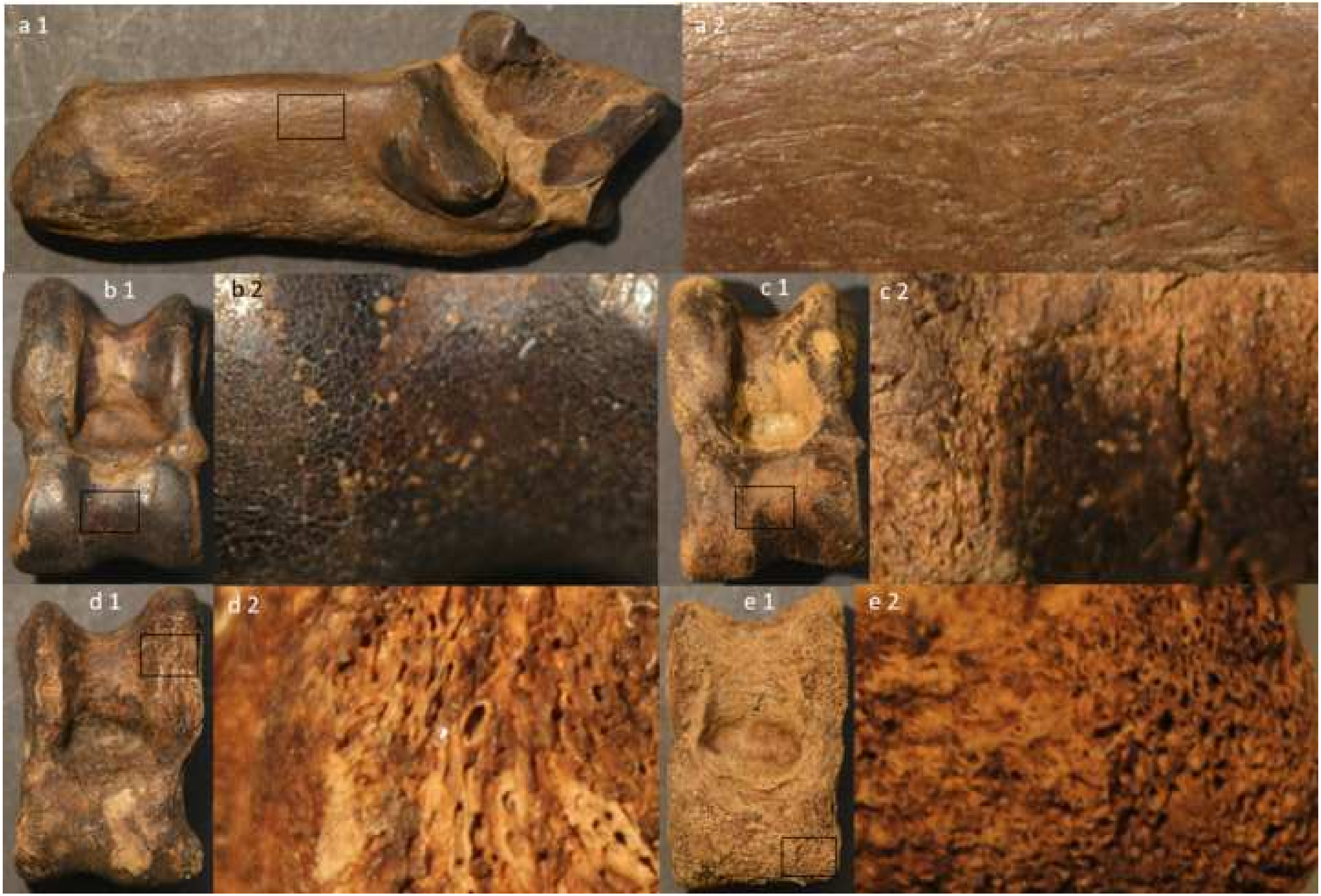

Figure

Click here to access/download;Figure;Fig. 7.tif

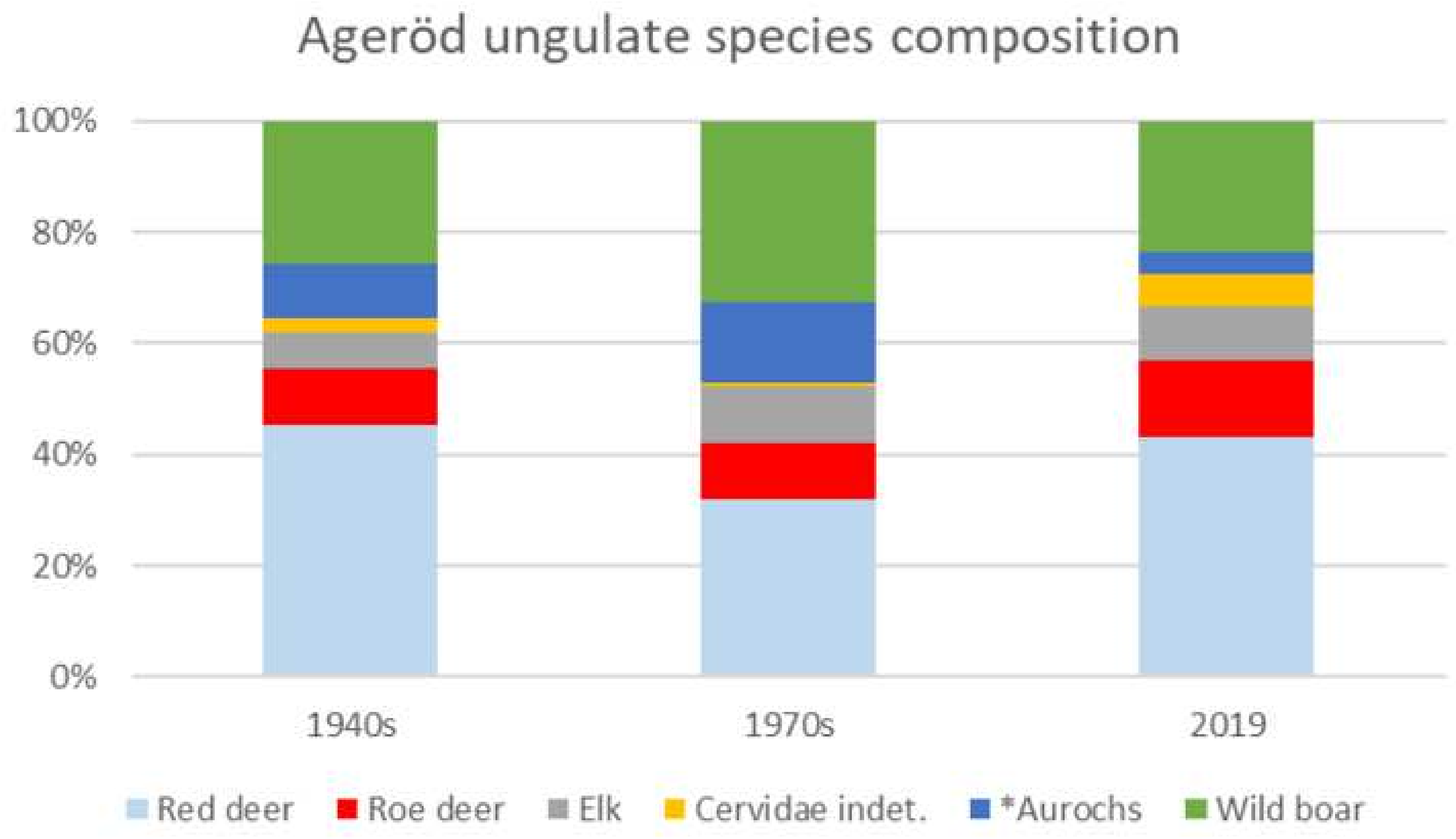

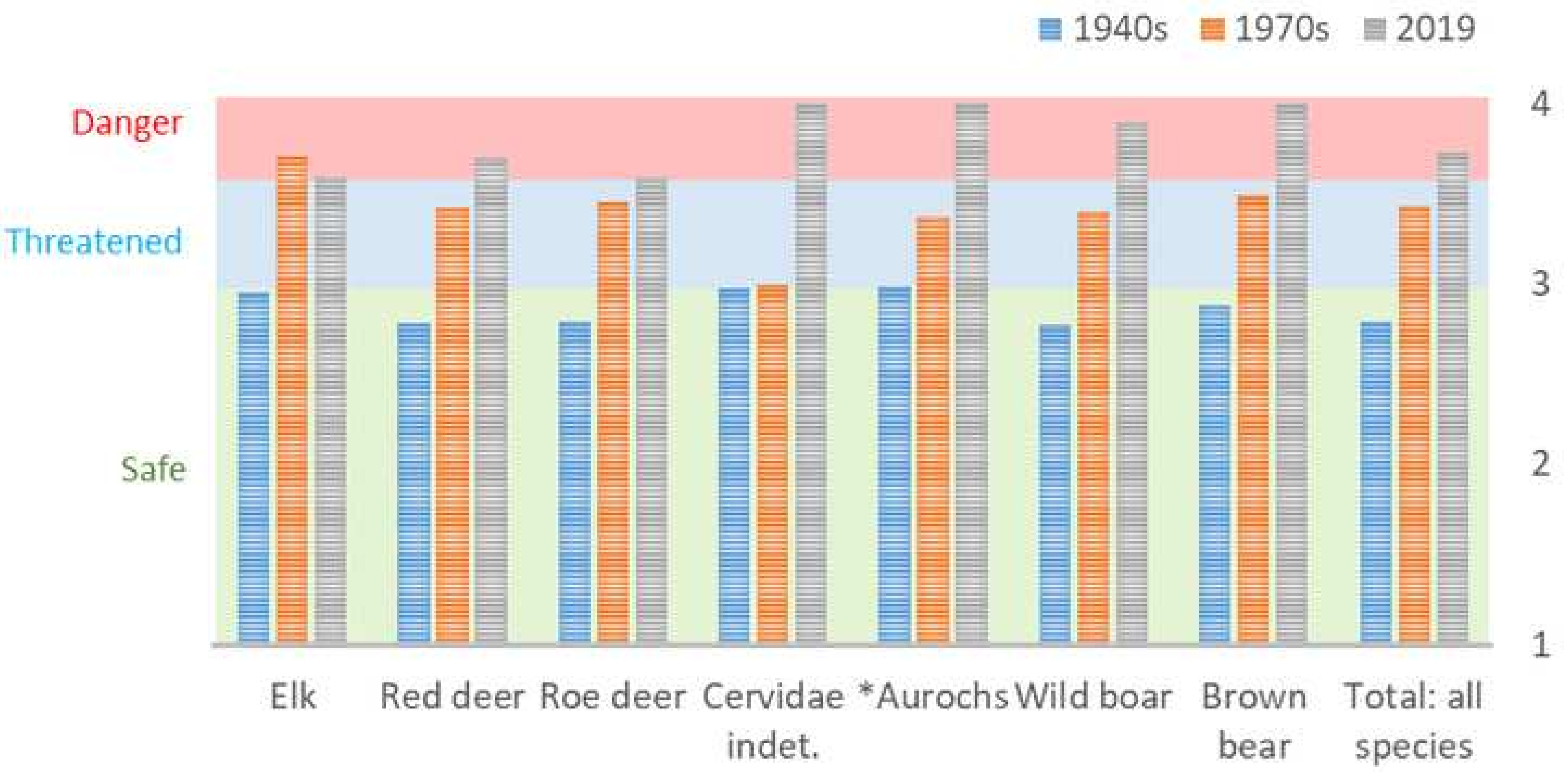
1940s
1970s
2019
Danger
Threatened
Safe
4
3
2
1
Elk
Red deer
Roe deer
Cervidae indet.
*Aurochs
Wild boar
Brown bear
Total: all species

Figure

Click here to access/download;Figure;Fig. 9.tif

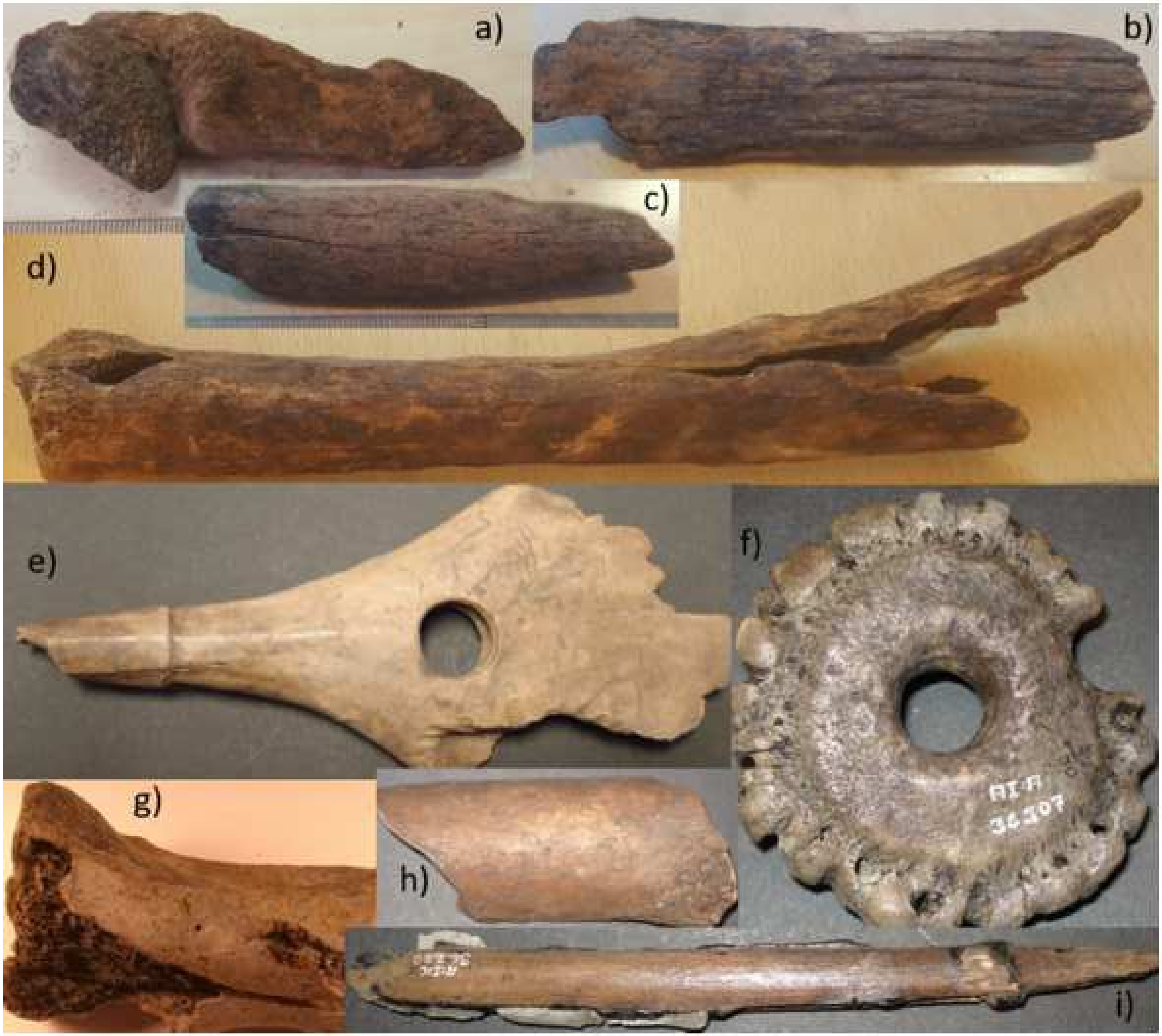

Figure

Click here to access/download;Figure;Fig. 10.tif

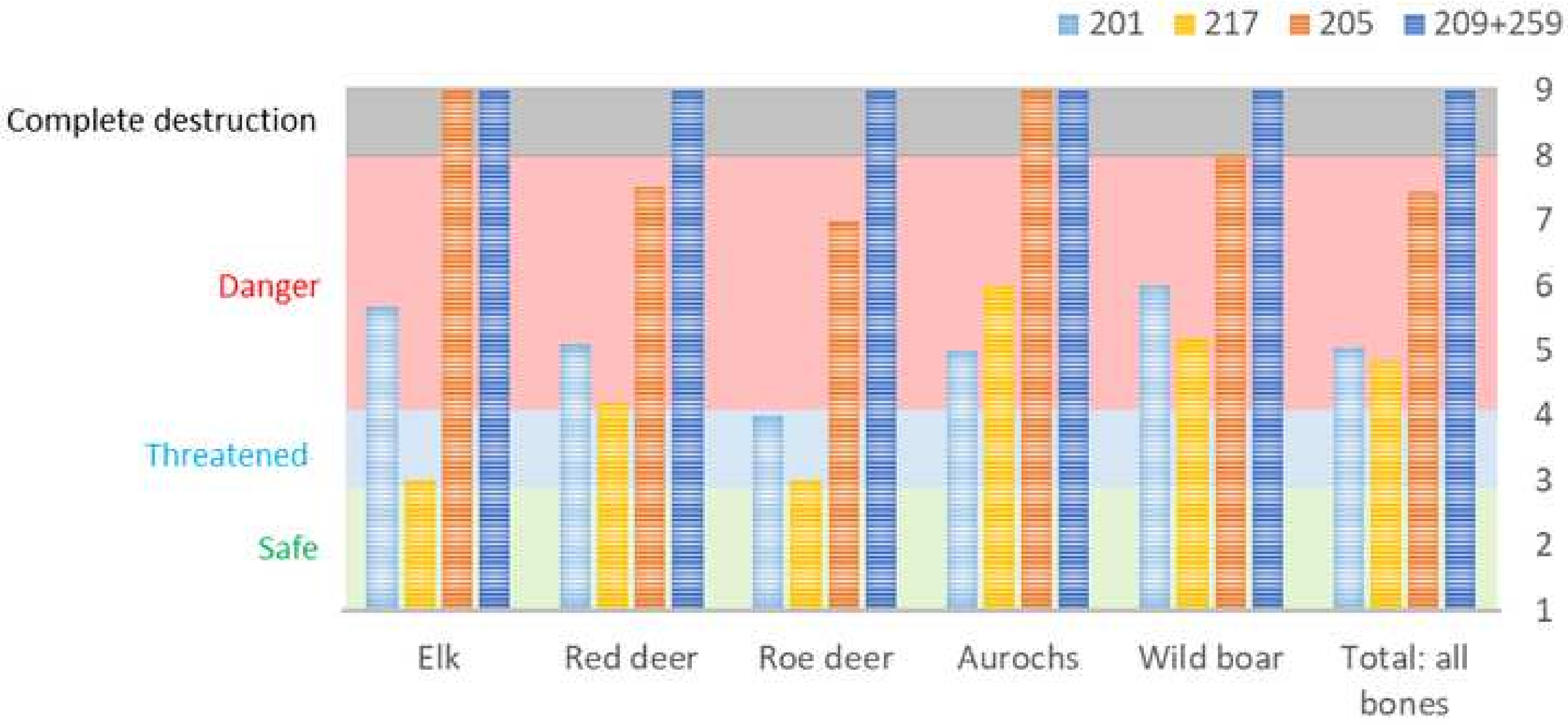

 Click here to access/download;Figure;Fig. 11.tif

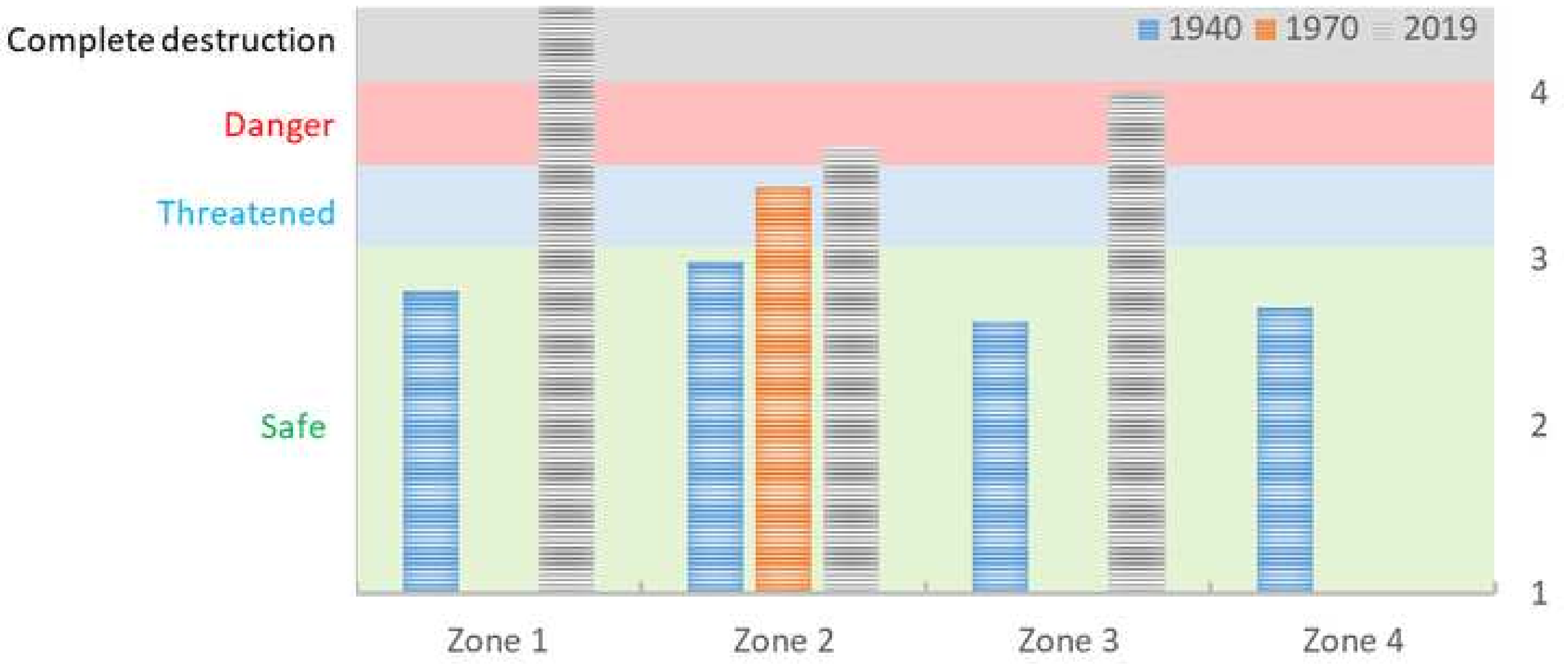

Figure

Click here to access/download;Figure;Fig. 12.tif

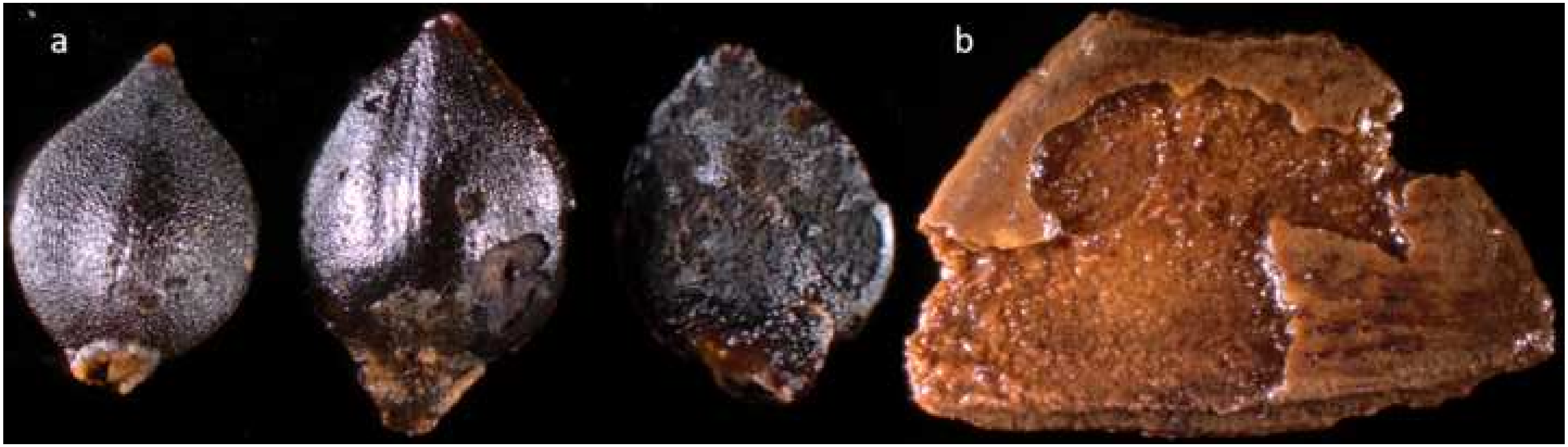

Click here to access/download
Supporting Information
renamed_2cae8.docx
