# Supplementary material for: Human encroachment, climate change and the loss of our archaeological organic cultural heritage: Accelerated bone deterioration at Ageröd, a revisited Scandinavian Mesolithic key-site in despair
Source: PLoS One. 2020 Jul 29;15(7):e0236105. doi: 10.1371/journal.pone.0236105 (PMC7390309; doi:10.1371/journal.pone.0236105)
Supplement: Attachment — Submitted filename: PONE review comments.docx [file pone.0236105.s003.docx]

5. Review Comments to the Author

Please use the space provided to explain your answers to the questions above. You may also include additional comments for the author, including concerns about dual publication, research ethics, or publication ethics. (Please upload your review as an attachment if it exceeds 20,000 characters)

Reviewer #1: This is a very important if deeply disturbing article, and parallels observations in the circumpolar north and high elevations. The news is very bad, but if it sparks large scale responses (and lots of rescue excavation) we have a chance to save something for the future. Very nice work, very frightening results. This needs immediate publication.

Reviewer #2: While I fully agree with the sentiment and broad conclusions of this paper, its presentation is currently not suitable for publication. It needs heavy and consistent language editing and some parts should, I suggest, be revised. This is true of both the conclusions drawn in relation to the data at hand - some statements are rather strong given the inherent uncertainties - while others are missed out on: this is particularly urgent with reagrd to the need for revised heritage management and financing aspects for 'climate rescue excavations'.

Toned down the parts deemed as too strong and included a small section of heritage management issues in the conclusion. Changed language according to suggestions by the reviewers and have done a final language revision after that.

I've tried to help with some of these aspects, so please see the comments in the annotated pdf.

Manuscript revised following comments in pdf and the changes are shown in the marked up copy of the manuscript.

Regarding the comment about the referred manuscript paper (line 362 & 384), we agree that it is not optimal to cite an unfinished manuscript. We have, however, stated the results from that paper in a clear way as to show that there can be no misinterpretations and they are part of a wider discussion and not the actual results presented in this paper. If we were to also include and show them here it would take the focus from what we are presenting here and because that manuscript paper includes histology, soil chemistry, collagen preservation and palaeobotanical analyses, it would double the length of this paper (which would not be optimal or reader friendly).

Finally, I just noticed that also the title might need revising (it's 'at' not 'on' Ageröd)

Fixed

Reviewer #3: Review PLOS ONE paper Boethius et al. Human encroachment…

Good article with important topic, but sometimes too focused on Swedish and/or Mesolithic material and publications. Quite a bit of work on in situ site preservation and changing preservation conditions has been carried out, particularly in the UK, DK, NL and Norway - some Mesolithic, many other periods, all relevant in this context. Some ref suggestions below.

minor language and typographical errors corrected (-> = change to)
JoAS: Journal of Archaeological Science; CMAS: Conservation and Management of Archaeological Sites
l. 22: unique, Done
l. 30: today if compared – delete if Done
l. 32: has not been -> has rarely been (there are refs on next page to published analyses) Done
l. 39: ago, Done
l. 96: Photography’s -> Photographs Done
l. 97: profile -> section Done
l. 119 70s -> 1970s Done and also updated elsewhere
l. 130: was done by -> was carried out by Done
l. 138: fixpoints in -> fixpoints from Done
l. 147: soil bank from when the ditch was dug and maintained throughout the last decades. -> soil bank of excavated ditch material from its establishment and maintenance. Done
l. 147: arbitrary -> arbitrarily Done
l. 153: excavation, Done
l. 154: layers -> deposits Done
l. 155: is -> are Done
l. 156: by layer, and all layers -> stratigraphically, and all deposits changed to “stratigraphically, and all depositional layers
l. 160, 165, 167, 290, 292, 372, 431: cultural layer(s) -> archaeological deposit(s). It is misleading to use the term deposits when we are discussing stratigraphically deposited layers. By more clearly stating “depositional layers” (see from previous comment) we think the term cultural layer is better for clarification we have used “white cultural layer” which is also compatible with previous publications of the site

l. 161: layers -> deposits Done
l. 164: Profiles -> Sections Done
l. 165: show -> shows Done
l. 178: surface, Done
l. 181, 182, 183: stage -> stages Done
l. 193: categories -> sub-categories Done
l. 212: constitute -> constitutes Done
l. 214: excavation, Done
l. 217: remains, Done
l. 238, 240: are -> is Done
Table 1, l. 290 (and all other use): Elk -> Moose (Elk= Cervus canadensis; Moose = Alces alces) No, we use the Euroasian term and not the North American

l. 248: excavation, Done
l. 252, 306: indicate -> indicates Done
l. 257, 258: is -> are Done
l. 265: excavation -> excavations Done
l. 272, 305, 325: increase -> increases Done
l. 298: was created -> were created Done
l. 307: appears -> appear Done
l. 309: delete when Done
l. 311: trench -> trenches Done
l. 319: numbers -> number Done
l. 331: were -> where Done
l. 338: who -> which Done
l. 340: focus -> focuses Done
l. 341: delete comma after analysis Done
l. 344: add reference, Boreham et al. 2011 JoAS 38, 2833-57 Done
l. 362: form -> forms Done
l. 377: add references to published material, eg. Gregory & Matthiesen 2012 CMAS 14, 479-86, Boreham et al. 2011 - possibly more; you are not the only ones to have come across this problem. Added “, which is a pattern also observed elsewhere (e.g. Borehamn et al., 2011)”
l. 426: cultural -> archaeological Done
l. 428: remains. ->add references: Martens et al. 2016 CMAS 18,8-29; Martens 2017 ARC 32.2, 123-140; Tjelldén et al. 2016 CMAS 18, 126-138 Added”( (for more intentional deterioration threat mitigation strategies see e.g. Martens et al. 2016; Martens 2017; Tjelldén et al. 2016)”
Fig. 4: Zone boarders -> Zone borders Done
Fig. 7, Fig. 10: Elk -> Moose *No, we use Euroasian term*
